# Genomic selection and genetic architecture of agronomic traits during modern flowering Chinese cabbage breeding

**DOI:** 10.1093/hr/uhae299

**Published:** 2024-10-18

**Authors:** Yahui Zhao, Guangguang Li, Zhangsheng Zhu, Ming Hu, Ding Jiang, Muxi Chen, Juantao Wang, Kexin Zhang, Yansong Zheng, Yi Liao, Changming Chen

**Affiliations:** Ministry of Agriculture and Rural Affairs Key Laboratory of South China Horticultural Crop Biology and Germplasm Enhancement, College of Horticulture, South China Agricultural University, Guangzhou 510642, China; Guangzhou Institute of Agriculture Science, Guangzhou 510308, China; Ministry of Agriculture and Rural Affairs Key Laboratory of South China Horticultural Crop Biology and Germplasm Enhancement, College of Horticulture, South China Agricultural University, Guangzhou 510642, China; Ministry of Agriculture and Rural Affairs Key Laboratory of South China Horticultural Crop Biology and Germplasm Enhancement, College of Horticulture, South China Agricultural University, Guangzhou 510642, China; Guangzhou Institute of Agriculture Science, Guangzhou 510308, China; Guangdong Helinong Biological Seed Industry Co., Ltd, Shantou, Guangdong 515800, China; Ministry of Agriculture and Rural Affairs Key Laboratory of South China Horticultural Crop Biology and Germplasm Enhancement, College of Horticulture, South China Agricultural University, Guangzhou 510642, China; Ministry of Agriculture and Rural Affairs Key Laboratory of South China Horticultural Crop Biology and Germplasm Enhancement, College of Horticulture, South China Agricultural University, Guangzhou 510642, China; Guangzhou Institute of Agriculture Science, Guangzhou 510308, China; Ministry of Agriculture and Rural Affairs Key Laboratory of South China Horticultural Crop Biology and Germplasm Enhancement, College of Horticulture, South China Agricultural University, Guangzhou 510642, China; Ministry of Agriculture and Rural Affairs Key Laboratory of South China Horticultural Crop Biology and Germplasm Enhancement, College of Horticulture, South China Agricultural University, Guangzhou 510642, China

## Abstract

Flowering Chinese cabbage is a type of leafy vegetable that belongs to the *Brassica* genus. Originally native to South China, it is now widely cultivated and consumed across the globe, particularly in Asian countries. The recent cultivation and regional expansion of flowering Chinese cabbage provides a valuable opportunity to elucidate the genomic basis underlying environmental adaptation and desired traits during a short-term artificial selection process. Here, we investigate the genetic variation, population structure, and diversity of a diverse germplasm collection of 403 flowering Chinese cabbage accessions. Our investigation seeks to elucidate the genomic basis that guides the selection of adaptability, yield, and pivotal agronomic traits. We further investigated breeding improvement associated with stem development by integrating transcriptome data. Genome-wide association analysis identified 642 loci and corresponding candidate genes associated with 11 essential agronomic traits, including plant architecture and yield. Furthermore, we uncovered a significant disparity in the allele frequency distribution of nonsynonymous mutations in these candidate genes throughout the improvement stages. Our results shed light on the genetic basis of improvement and crucial agronomic traits in flowering Chinese cabbage, offering invaluable resources for upcoming genomics-assisted breeding endeavors.

## Introduction

Since the dawn of agricultural civilization ~10 000 years ago, wild plants have undergone successful domestication and improvement through continuous artificial selection to meet human needs [1, 2]. Monitoring key molecular changes in this process is crucial for guiding further improvements or the *de novo* domestication of crops [2, 3]. However, the breeding of numerous crops predominantly depends on experiential knowledge and manual observation, thereby limiting the breeding process. The rapid advancement of sequencing technologies, especially next-generation sequencing (NGS), has transformed the tracking of genome-wide selection signatures and their application in crop improvement, as reported in rice, maize, and rapeseed, among others [4–7]. Employing high-throughput genomic and phenotypic datasets, genome-wide association studies (GWASs) have identified loci or genes associated with key agronomic traits in diverse species [4, 7–10]. This development has facilitated the implementation of marker-assisted selection and molecular design breeding, thereby accelerating the pace of breeding initiatives.

*Brassica rapa*, an important member of the *Brassica* genus ‘triangle of U’ model, has formed into multiple subspecies and variants during the diversification process with diverse morphotypes, such as leafy heads, enlarged organs, and extensive axillary branching [11]. This diversity has made it a hot topic in plant science research on crop domestication and morphological differences. Previous studies on *B. rapa* domestication origins and intraspecific diversity have been extensively conducted through whole-genome resequencing, revealing the crucial role of parallel selection at the subgenome level in morphotype diversification [12, 13]. For the study of selective processes in modern breeding, one case is the resequencing of 194 different ecotypes of Chinese cabbage [14]. This study elucidated the geographic origins and selective dynamics of spring Chinese cabbage and identified that the sequence variation on the *BrVIN3.1* promoter contributes to differences in vernalization response among various ecotypes.

Flowering Chinese cabbage (*B. rapa* ssp*. chinensis* var*. parachinensis*), also known as Caixin, is a variant of pak choi originating from southern China [11, 12, 15]. This vegetable possesses significant economic and health value [16–19]. In recent decades, deliberate breeding has markedly improved its adaptability, yield, and quality, leading to extensive cultivation throughout northern and southern China [20, 21]. Despite these advancements, the increasing consumer demand and the need for expanded cultivation areas present ongoing challenges. Currently, the breeding practices of flowering Chinese cabbage primarily rely on traditional methods and cross-breeding, which are time-consuming and cannot improve multiple traits simultaneously [20, 21]. Recent studies suggest that molecular breeding techniques hold substantial potential for accelerating trait improvement and developing new varieties of flowering Chinese cabbage [15, 22, 23]. Thus, understanding the genetic basis for the improvement is crucial, yet in-depth research on this topic remains limited.

The recent release of the high-quality reference genome, Youlv 701, provides a robust foundation for our research on flowering Chinese cabbage [24]. Through whole-genome resequencing of 403 flowering Chinese cabbage germplasms representing a diversity of breeding periods, combined with phenotypic and transcriptomic data, this study comprehensively assessed the genomic variations, population structure, and the genetic basis of both modern breeding improvements and important traits in the flowering Chinese cabbage population. Our objective was to reveal the population characteristics of flowering Chinese cabbage, clarify improvement signals of modern breeding processes, and identify specific loci or genes associated with 11 agronomic traits. Moreover, by using the linkage disequilibrium (LD) of trait-related loci, this study constructed the possible genetic network that regulates these 11 traits. Our research reveals the genetic factors critical to the structure, adaptability, and yield of flowering Chinese cabbage, providing valuable insights for plant molecular breeding.

## Results

### Genome sequence, genetic diversity, and population structure

To explore the flowering Chinese cabbage’s genetic diversity and population structure, we sequenced the genomes of 403 genotypes representative of breeding periods (i.e. early landraces, modern elite cultivars, and improved inbred line), using 150 bp paired-end sequencing technology (Supplementary Table S1). This approach generated 1.2 terabytes of sequencing data, with an average mapping rate of 98.3% and ~6.7× genomic coverage (Supplementary Table S2). By mapping these short reads to the Youlv 701 reference genome, we identified 2 515 078 high-confidence single-nucleotide polymorphisms (SNPs) and 656 098 insertions/deletions (InDels), with a minor allele frequency (MAF) exceeding 5% and a missing rate below 20% (Supplementary Fig. S1). Among these SNPs, 28.9% (729 170/2515078) overlapped with genic regions. The ratio of nonsynonymous to synonymous substitutions stood at 0.45, akin to Chinese cabbage (0.42), yet lower than *B. rapa* (0.54), indicating relatively narrow genetic variation within this variety (Supplementary Table S3) [12, 14].

**Figure 1:**
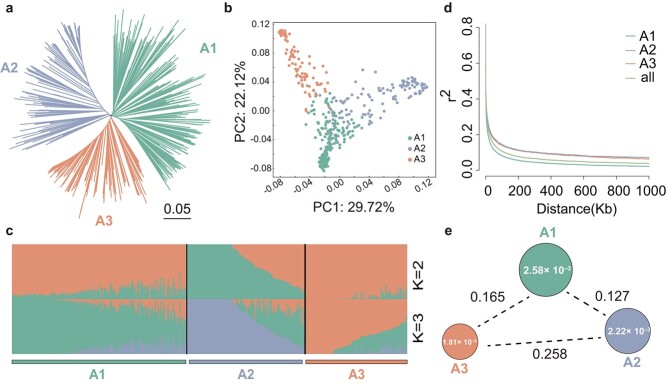
Population structure and genetic divergence of 403 flowering Chinese cabbage accessions. (a) Phylogenetic tree of all accessions constructed from the whole-genome SNPs. The 403 accessions are classified into three subgroups. (b) Principal component analysis plot of all the accessions used in this study. (c) Population structure of the flowering Chinese cabbage accessions for *K* = 2–3. (d) Decay of LD in each population. (e) Nucleotide diversity (π) and population differentiation (F_ST_) among three subgroups. The value in each circle indicates the level of nucleotide diversity (π) for each population, and the value on each line represents population divergence (F_ST_) between the two populations.

Based on the neighbor-joining (NJ) phylogenetic tree constructed from SNPs, 403 samples were divided into three subgroups: landraces (A1) and two improved germplasms (A2, A3) (Fig. 1, Supplementary Fig. S2). Group A1 mainly consists of landraces with local names and early commercial varieties, like 49-19 (1970s), while Groups A2 and A3 predominantly contain modern commercial varieties and improved inbred lines designated by numbers, such as Loulv701 (2005). This classification was supported by the principal component analysis and population structure analysis (*K* = 3). In addition, the genetic relation kinship analysis also showed that each subgroup had relatively higher genetic similarity (Supplementary Fig. S2b). To further elucidate the relationships among these groups, we constructed a phylogenetic tree with turnip as the outgroup [11]. The findings showed that local varieties A1 were more closely related to ancestral roots, whereas the improved variety A3 exhibited the greatest level of differentiation (Supplementary Fig. S2c).

To assess the genetic diversity and divergence among the subgroups of flowering Chinese cabbage, we calculated the nucleotide diversity (π) and pairwise fixation index (F_ST_) for each population (Fig. 1e). Genetic diversity showed a decreasing trend from the landraces A1 (π = 2.58 × 10^−3^) to the improved populations A2 (π = 2.22 × 10^−3^) and A3 (π = 1.81 × 10^−3^), with an overall genetic diversity of 2.6 × 10^−3^. The above results indicate that subgroups A1–A3 may sequentially depict the transition from traditional landraces to recent modern cultivars. Notably, a considerable portion of genetic diversity was retained throughout the improvement process, indicating a relatively weak improvement bottleneck in flowering Chinese cabbage (π_landrace/π_improvement = 1.16–1.43). Additionally, comparisons between the landrace species and the two improved species (*F*_ST_ = 0.127–0.165) revealed greater genetic differentiation in the improved species (*F*_ST_ = 0.258). We hypothesize that this disparity stems from population differentiation linked to specific breeding objectives pursued during various breeding periods.

Further genetic analysis revealed varying linkage disequilibrium (LD) levels across the subgroups. The LD decay distance increased gradually from the landraces A1 (15 kb, *r*^2^ = 0.2) to improved varieties A2 and A3 (32.4–36.8 kb, *r*^2^ = 0.2) (Fig. 1d). Consequently, the overall LD decay distance averaged ~23.2 kb (~1.8 kb, half maximum *r*^2^). These findings suggest that modern improved varieties have experienced stronger selective pressures compared to the landraces, aligning with the observed decline in genetic diversity. Additionally, the study revealed that the LD decay distance in flowering Chinese cabbage is less than that of Chinese cabbage (4.98 kb, half maximum *r*^2^) and *B. rapa* (2.9 kb, half maximum *r*^2^) [12, 14]. This relatively rapid LD decay implies that association studies can attain higher resolution, thereby facilitating the identification of narrow candidate quantitative trait loci (QTL) intervals.

### Phenotypic variation, heritability, and correlation in flowering Chinese cabbage

The flower stalks and leaves, as the main nutritional and edible organs of flowering Chinese cabbage, not only reflect the overall growth condition of the plant but also determine its commercial value, making them a primary target for breeding improvements. Over three consecutive years (2019–21), we evaluated 11 related agronomic traits of flowering Chinese cabbage in a collection of 403 germplasm accessions. These traits include plant height (PH), plant breadth (PB), basal leaf number (BLN), characteristics of the maximum basal leaf [leaf length (LL), leaf width (LW), petiole length (PL), petiole width (PW)], weight per plant (WPP), length of main flower stalk (LFS), basal diameter of main flower stalk (DFS), and weight of main flower stalk (WFS) (Supplementary Fig. S3a). A detailed description and evaluation of these traits is provided in Supplementary Table S4. Our results revealed a large variation of these 11 traits in the studied population. For instance, WPP varied from 10.1 to 213 g and WFS from 2.88 to 128 g across all accessions, exhibiting nearly 20- and 40-fold differences, respectively. The coefficients of variation (CVs) for WPP and WFS were 51.68%–59.69% and 50.57%–64.75%, respectively, over the 3-year period, with PL also showing larger CV values (33.95%–40.38%). This indicates the potential of these traits in selecting high-yield and diverse varieties of flowering Chinese cabbage. Conversely, the PH, PB, and LL displayed minimal phenotypic variation, with the CV ranging from 17.83% to 20.92%. Heritability analysis revealed that leaf-related traits (LL, LW, PL, PW), WPP, and DFS had relatively high broad-sense heritability, ranging from 66.42% to 79.84% (Supplementary Fig. S3c). This underscores their genetic stability, necessitating less environmental variation and repetition for evaluating their applicability in screening superior varieties. In contrast, BLN, LFS, and PB showed lower broad-sense heritability (30.78%, 43.64%, and 45.48%, respectively), indicating the necessity for multiyear replicates to effectively evaluate these traits.

The Best Linear Unbiased Estimation (BLUE) is a widely employed statistical method that improves the accuracy and reliability of genetic analysis by reducing the effects of measurement errors and environmental variations on phenotypic data [25]. In this study, we conducted a correlation analysis using the BLUE values of the 11 traits to elucidate their interrelationships. The findings revealed extensive positive correlations among these traits (Supplementary Fig. S4). LL and PL exhibited the strongest correlation (*r* = 0.91), followed by a robust positive correlation among PW, WPP, DFS, and WFS (*r*^2^ > 0.8). These findings highlight the strong correlation between yield and plant morphological traits. Additionally, strong positive correlations were also observed between PH and LFS (*r*^2^ = 0.88) and between PW and LL (*r*^2^ = 0.74). However, BLN did not show a substantial correlation with the other traits. The results of cluster analysis supported the above findings, grouping 11 traits into four groups (Supplementary Fig. S3b). The analysis above indicates that the flowering Chinese cabbage population possesses diverse phenotypic characteristics and genetic stability. It also reveals the connection between these traits, thereby enabling their utilization in predicting yield.

### Genomic signals associated with artificial selection at different breeding periods

**Figure 2:**
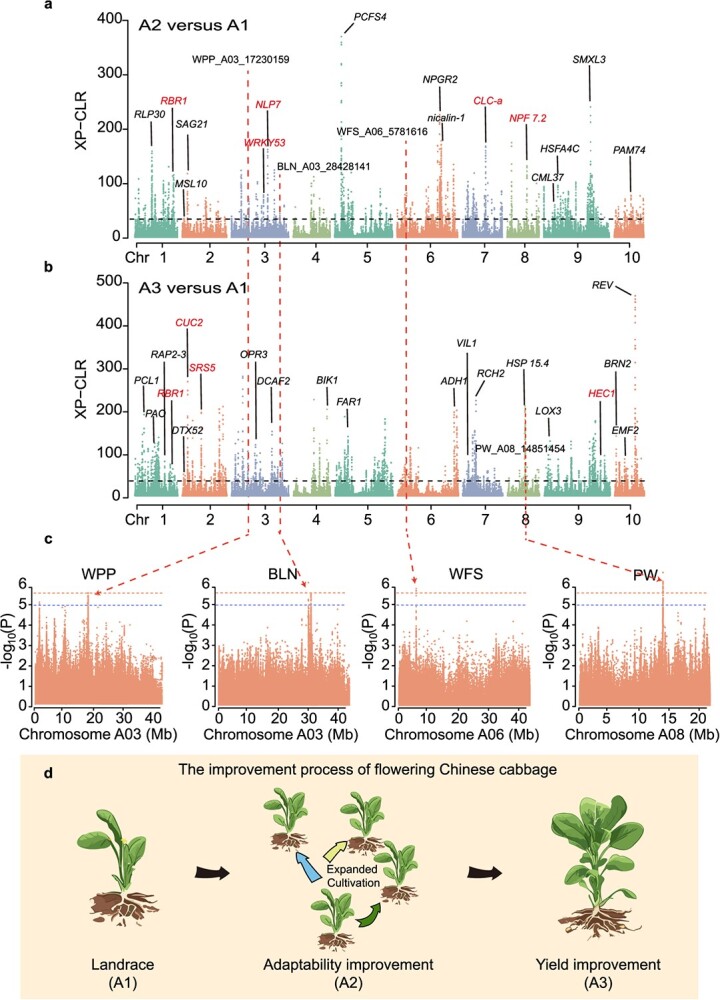
Genome-wide analysis of selective sweeps during modern flowering Chinese cabbage breeding. (a, b) Genome-wide selective signals between Landrace (A1) and Improved (A2, A3). The horizontal dashed lines indicate the cutoffs, with the highest being 1%. The annotated genes have been functionally characterized, and color annotation is the focus of attention (in red). (c) Manhattan plots of GWAS signals overlapping with the identified selective sweeps. The horizontal dashed line represents the significance threshold [*P <* 1.46 × 10^−6^ (red), *P <* 1 × 10^−5^ (blue)]. (d) Schematic diagram of modern breeding improvement process of flowering Chinese cabbage. The breeding practice of flowering Chinese cabbage aims to adapt it to a wider range of cultivation environments (A2) and further increase its yield (A3).

Since the 1960s, breeders have extensively studied the genetic improvement in flowering Chinese cabbage. This continuous breeding effort has resulted in the development of numerous new varieties, leading to ongoing improvements in both the planting area and yield of flowering Chinese cabbage. By the 1990s, the production of flowering Chinese cabbage had expanded to northern China, significantly improving the adaptability and agronomic traits of the crop through continuous selection [20, 21]. Moreover, different populations of flowering Chinese cabbage exhibit distinct morphological characteristics, which may result from continuous breeding selection (Supplementary Fig. S5). This serves as an ideal model for understanding the genomic basis underlying these selection processes. To identify potential selection signals associated with the modern improvement of flowering Chinese cabbage, we utilized the cross-population composite likelihood ratio (XP-CLR), π ratio tests, and *F*_ST_ methods across different breeding eras (Fig. 2, Supplementary Fig. S6, and Supplementary Tables S5 and S6). The findings suggest that flowering Chinese cabbage breeding has undergone two main stages: improvement in environmental adaptability and yield increase (Fig. 2d).

The first stage primarily involves adapting to the environment. In subgroup A2, we employed a combination of F_ST_-π ratio and XP-CLR analyses to identify 377 and 3384 potential selection signals, respectively, which collectively cover 1.5% (5.48 Mb) and 2.9% (10.97 Mb) of the assembled genome, corresponding to 831 and 1664 genes (Fig. 2a, Supplementary Figs S6a and S7a, and Supplementary Tables S5–S7). Among these, 246 genes were consistently identified. Gene Ontology (GO) enrichment analysis indicated that these genes were enriched in various terms associated with adaptation to oxidative stress and nitrogen nutrition, including 'response to reactive oxygen species', 'response to nitrate', and 'programmed cell death in response to reactive oxygen species' (Supplementary Fig. S7c). Among the identified genes, *NLP7, CLC-a*, and *NPF7.2* were related to nitrate uptake and transport, whereas *SAG21, SMXL3, HSFA4C*, *CML37*, and *PAM74* were linked to environmental response and signal transduction (Fig. 2a, Supplementary Table S7) [26–28]. Furthermore, genes related to 'leaf senescence' and 'chlorophyll biosynthesis', such as *SPH3, MSL10,* and *WRKY53,* were also highlighted as participating in adaptive improvements.

The second stage aims to improve yield. During the high-yield selection process, we noticed significant changes in these traits. Apart from LW, all other traits exhibited a notable increase overall (Supplementary Fig. S5). In subgroup A3, 670 and 3371 selection signals were identified, which accounted for 2.4% (8.9 Mb) and 2.9% (10.9 Mb) of the assembled genome, respectively. These signals encompassed 1133 and 1741 genes, respectively, with 543 genes consistently identified (Fig. 2b, Supplementary Figs S6b and S7a and Supplementary Tables S5–S7). Notably, these genes were mainly linked to plant growth and developmental processes, including 'shoot system development', 'phyllome development', and 'plant organ development' (Supplementary Fig. S7d). Key genes involved in plant organ morphogenesis, such as *RBR1, CUC2, DCAF1, RCH2, EMF2*, and *PLL5*, were identified among the selected genes (Fig. 2b and Supplementary Table S7) [29–32]. Additionally, 250 genes were consistently identified at two stages (Supplementary Fig. S7a and Supplementary Table S7). These genes were significantly enriched in terms related to environmental adaptation, such as 'leaf senescence', 'short photoperiod', and 'vernalization response' (Supplementary Fig. S7e). These results suggest that genes related to environmental adaptation may have experienced multiple rounds of selection.

In order to further elucidate the genetic mechanisms of yield improvement, we integrated available transcriptome data representing different developmental stages of flowering Chinese cabbage stalks. This led to the identification of 389 differentially expressed genes that overlapped with yield improvement signals (Supplementary Fig. S7b and Supplementary Table S8) [33]. Among these, up-regulated genes are mainly associated with plant metabolic processes, enzyme activities, and morphogenesis, whereas down-regulated genes are enriched in terms related to plant environmental adaptation, hormone metabolism and regulation, cell signal transduction, and cell structure formation (Supplementary Fig. S7f and g). These genes play crucial roles in various aspects of plant growth (Supplementary Tables S7 and S8). For instance, *MIOX2, XTH31, CESA2, CASP5*, and *4CL3* regulate cell wall composition, encompassing cellulose and lignin synthesis, while *CYP707A1, NCED4, LECRK41, HEC1*, and *SRS5* participate in the hormone metabolic pathway. Additionally, genes like *CUC2, HERK1*, and *FH1* are associated with plant organ development, such as cell elongation.

### Elite-allele frequency under selection during breeding

Nonsynonymous mutations could cause the amino acid composition of proteins encoded by genes, frequently influencing gene function and expression, which, in turn, can impact the agronomic traits of plants [7]. Consequently, variations in the frequency of related alleles can serve as indicators of selection trends in breeding. We analyzed nonsynonymous SNPs in genes potentially selected over various breeding periods (Supplementary Fig. S8). The findings reveal that these SNPs underwent convergent selection, suggesting substantial selection pressure during breeding. Significant differences in allele frequencies were found in these genes during modern breeding. For example, the allele frequency of genes such as *WRKY53* and *PWR*, regulators of leaf senescence, and *NLP7* and *CLC-A*, responsible for nitrate assimilation and signal transduction, was markedly increased during the adaptive improvement phase (A2) (Supplementary Fig. S8a). The allele frequency of genes related to plant growth and morphogenesis, such as *CUC2, DCAF1, RCH2, EMF2*, and *PLL5*, was significantly increased in the yield improvement stage (A3) (Supplementary Fig. S8b). In addition, genes involved in vernalization and photoperiod pathways such as *BRN2, VIL1, PCL1*, and the hormone regulator during reproductive organ development *HEC1* showed significant frequency changes in both improvement stages. These findings align with the historical breeding patterns of flowering Chinese cabbage, suggesting that advantageous alleles are likely to be selected and retained during the breeding improvement.

### Genome-wide association studies for 11 agronomic traits

**Figure 3:**
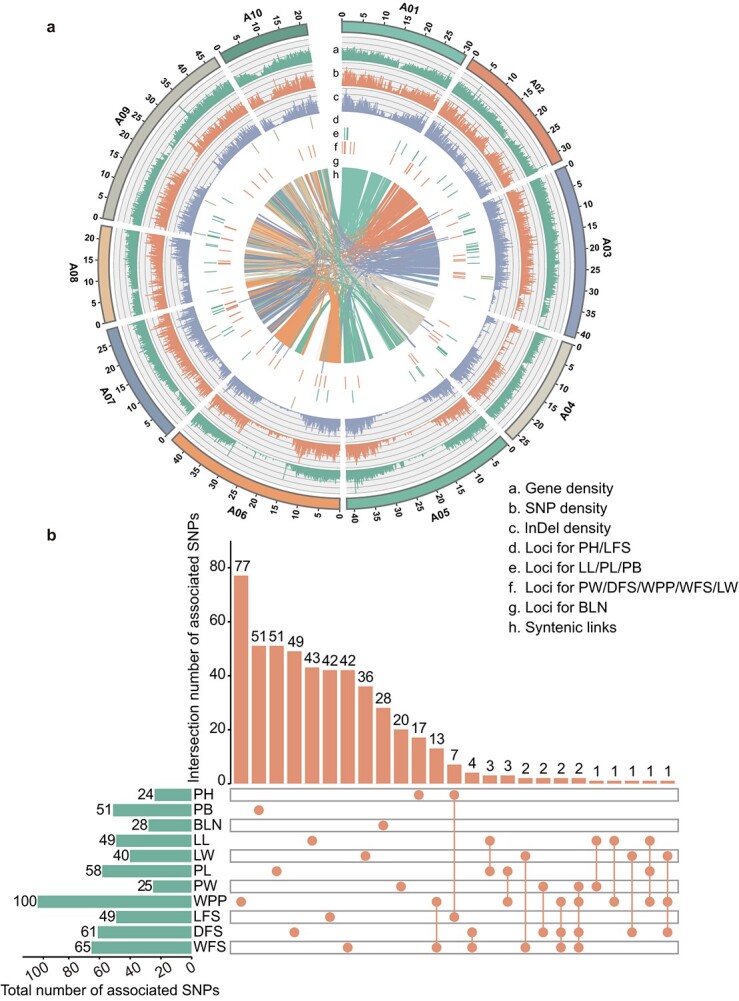
Genome-wide association study of 11 agronomic traits. (a) The Circos plot showing features of flowering Chinese cabbage genome and loci associated with the 11 agronomic traits. The Manhattan plots of each agronomic trait are shown in Supplementary Fig. S9. (b) Upset Venn diagram showing the overlap number of the SNPs associated with the 11 agronomic traits. The horizontal histogram at the left shows the total number of SNPs in each agronomic trait. The vertical histogram at the top shows the number of unique and common SNPs. The dots represent the presence of the SNPs associated with the trait that is listed on the left side. The vertical column with only one dot represents unique SNPs in one trait. The vertical column with more than one dot represents the common SNPs for multiple traits.

Based on the obtained 2 515 078 SNPs and 656 098 InDels, GWAS analysis was performed on the BLUE values of 11 traits further to understand the genetic basis of flowering Chinese cabbage traits (Fig. 3). Using various methods, a total of 499 trait-related SNPs were identified. Among these, 115 were identified by more than two methods, 43 were consistently associated with multiple traits, and 7 were consistently detected by multiple traits and methods as key candidate loci (*P <* 1 × 10^−5^, Fig. 3, Table 1, Supplementary Fig. S9, and Supplementary Table S9). Additionally, InDel-GWASs identified 143 InDels for the 11 traits, with 108 loci overlapping with SNP-GWAS signals and the rest detected only in InDel-GWASs, indicating the complementarity of InDel-GWASs as a simple and effective method for identifying candidate genes associated with traits (*P <* 1 × 10^−5^, Supplementary Fig. S10 and Supplementary Table S10). By merging the significant locus with the LD decay distance of the flowering Chinese cabbage population, a candidate interval was formed. Ultimately, 2608 candidate genes were obtained for the 11 traits (Supplementary Table S11). Additionally, 84 SNPs and 29 InDels were found to overlap with the signal regions of improvement, indicating that potential genes or loci associated with these traits may contribute to modern breeding advancements (Fig. 2c, Supplementary Table S12).

**Table 1:** Comprehensive summary of GWAS-identified candidate genes.

**Trait**	**GWAS loci**	**Candidate gene**	**Description**
PH, LFS	A02–30595991	*Bra_cxA02g003580*	PLASMODESMATA CALLOSE-BINDING PROTEIN 1(*PDCB1*)
PH	A04–14791097	*Bra_cxA04g020510*	Mitogen-activated protein kinase kinase 3(*MKK3*)
LL, PL	A03–28137709	*Bra_cxA03g011540*	Casein kinase 1-like protein 3 (*CKL3*)/TCP interactor containing EAR motif protein 1 (*TIE1*)
		*Bra_cxA03g011560*	Methyltransferase-like protein 5
PW, DFS, WFS, WPP	A09–1668557	*Bra_cxA09g068050*	STRUBBELIG-RECEPTOR FAMILY 3 (*SRF3*)
LW, DFS, WFS, WPP	A01–2181079	*Bra_cxA01g042490*	DEFECTIVE EMBRYO AND MERISTEMS 1(*DEM1*)
PW, WFS, WPP	A02–1569970	*Bra_cxA02g046520*	Probable LRR receptor-like serine/threonine-protein kinase
DFS, WFS	A07–24297027	*Bra_cxA07g009110*	PLASMODESMATA CALLOSE-BINDING PROTEIN 4 (*PDCB4*)
PW, DFS	A08–14851454	*Bra_cxA08g017120*	ATP-dependent DNA helicase
WFS, WPP	A03–17230159	*Bra_cxA03g031190*	Transducin/WD40 repeat-like superfamily

#### Plant architecture

**Figure 4:**
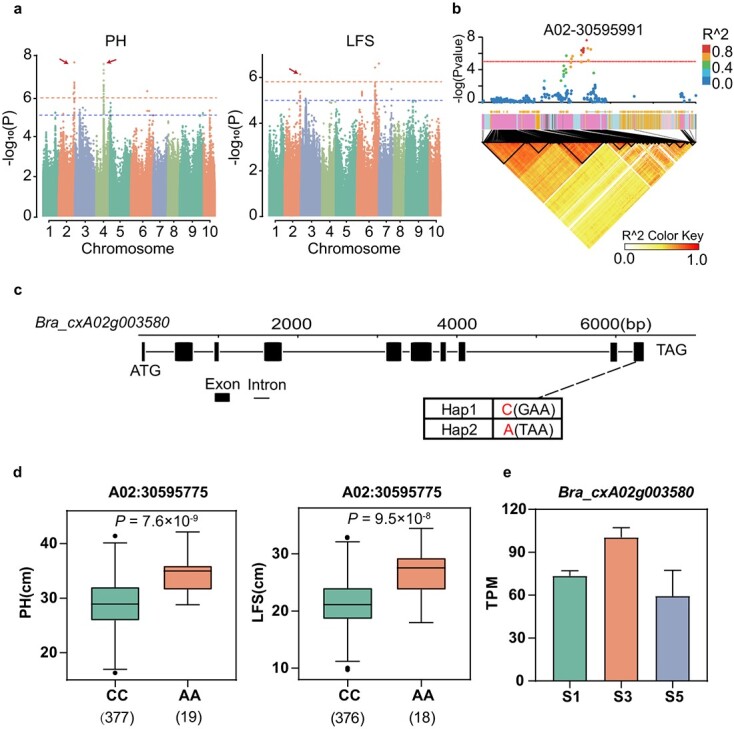
GWAS for plant height, length of the main flower stalk, and identification of the candidate gene *Bra_cxA02g003580*. (a) Manhattan plots showed the locus (A02:30595991, A04:14791097) for PH and LFS based on SNP-GWAS. (b) Local Manhattan plot (top), gene models (middle), and LD heatmap (bottom) surrounding the A02-30595991. The horizontal dashed line represents the significance threshold (*P <* 1 × 10^−5^). (c) Exon-intron structure and DNA polymorphism of *Bra_cxA02g003580*. (d) Boxplot of PH, LFS for the haplotypes (Hap) of *Bra_cxA02g003580*. Center line, median, box limits, upper and lower quartiles; whiskers, 1.5× the interquartile range; and dots represent outliers. Significant differences between the haplotypes were evaluated by a two-tailed *t*-test and shown by *P*-value or different letters (*P <* 0.05). (e) Expression levels of *Bra_cxA02g003580* in different stages of stalks based on TPM from RNA-seq results. S1, S3, and S5 represent the seedling stage, the bolting stage, and the flowering or harvesting stage, respectively.

Improving stalk-related traits is crucial for increasing yield and optimizing cultivation management in flowering Chinese cabbage, a typical stalk vegetable [33]. The GWAS analysis identified 24 SNP loci associated with PH and 49 SNP loci correlated with LFS (Supplementary Tables S9). Notably, the SNP A02-30595991, located within the gene *Bra_cxA02g003580* that encodes PLASMODESMATA CALLOSE-BINDING PROTEIN 1 (*PDCB1*), was repeatedly associated with PH and LFS (Fig. 4a, Table 1, *P =* 2.44 × 10^−8^). Moreover, the strongest association SNP for PH was A04-14791097 (*P =* 1.45 × 10^−10^), which is within the gene *Bra_cxA04g020510* encoding Mitogen-activated protein kinase kinase 3 (*MKK3*). Further analysis indicated that these two genes were upregulated during the bolting stage, and mutations in them exhibited a high correlation with the corresponding traits (*P <* 5.8 × 10^−7^), suggesting their potential role as candidate genes in regulating these traits (Fig. 4, Supplementary Fig. S11).

**Figure 5:**
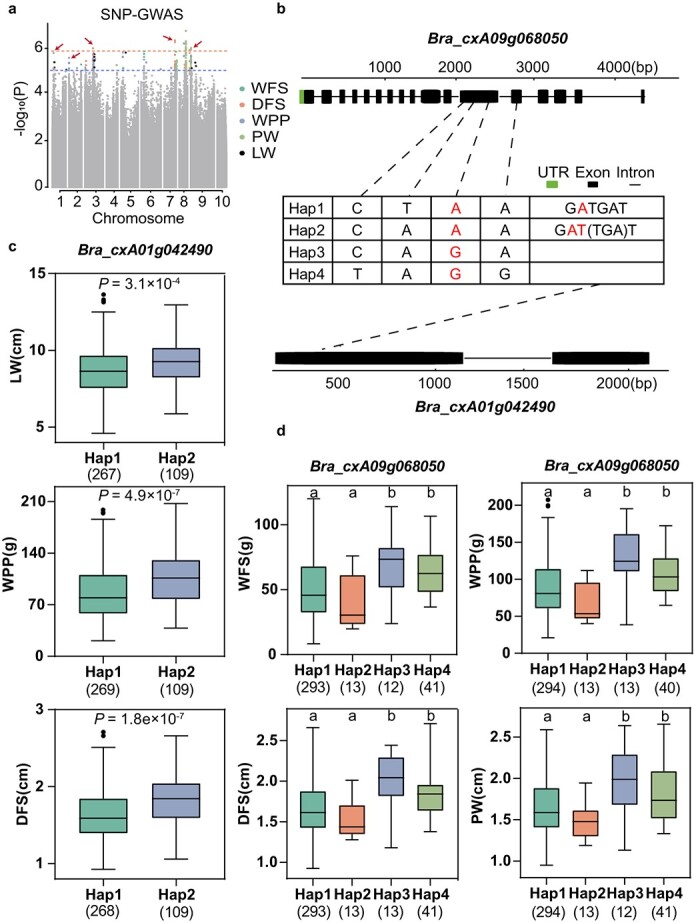
GWAS for yield-related traits and identification of the candidate genes *Bra_cxA09g068050* and *Bra_cxA01g042490*. (a) Manhattan plots showed the overlapping locus for LW, PW, WFS, DFS, and WPP based on SNP-GWAS. (b) Exon–intron structure and DNA polymorphism of *Bra_cxA09g068050* and *Bra_cxA01g042490*. (c, d) Boxplot of LW, PW, WFS, DFS, and WPP for the haplotypes (Hap) of *Bra_cxA09g068050* and *Bra_cxA01g042490*. Center line, median, box limits, upper and lower quartiles; whiskers, 1.5× the interquartile range; and dots represent outliers. Significant differences between the haplotypes were evaluated by a two-tailed *t*-test and shown by *P*-value or different letters (*P* < 0.05).

LL and PL are important traits to assess plant structure and growth ability, and they are highly correlated [34]. Therefore, the study highlighted SNP A03-28137709 for its consistent association with both traits (Supplementary Fig. S12, Supplementary Table S9). Two expressed genes, *Bra_cxA03g011560* and *Bra_cxA03g011540*, near this locus encode Methyltransferase-like protein 5 and *CKL3/TIE1*, respectively (Table 1). These genes were significantly linked to the traits, with their haplotypes exhibiting marked variations throughout different breeding periods (Supplementary Fig. S12c and d). Furthermore, *Bra_cxA03g011560* may interact with *TRM112A* and *TRM112B*, regulatory factors of cell division in organ growth processes (Supplementary Fig. S12e). On the other hand, *Bra_cxA03g011540* is implicated in plant blue light response (*CKL3*) and leaf development (*TIE1*), exhibiting high expression levels at different leaf developmental stages (Supplementary Fig. S12f) [35]. Thus, these candidate genes may play roles in leaf development, thereby regulating LL and PL.

#### Yield-related traits

Yield, a complex and crucial trait, is a primary objective in the breeding of flowering Chinese cabbage. Phenotypic analysis has identified LW, PW, DFS, WFS, and WPP as the main components contributing to yield, all of which display strong positive correlations (Supplementary Figs S3 and S4). Notably, SNP A09-1668557, located in the gene *Bra_cxA09g068050*, is associated with DFS, WFS, PW, and WPP (*P =* 1.74 × 10^−13^, Fig. 5, Table 1). This gene encodes the protein STRUBBELIG-RECEPTOR FAMILY 3 (*SRF3*), which is highly expressed in the early development of plant organs (Supplementary Fig. S13b). Nonsynonymous SNPs in this gene significantly influence expression levels and phenotypes (*P <* 0.05), which may indicate a role in regulatory processes affecting these yield-related traits (Fig. 5b and d, Supplementary Fig. S13c).

Another noteworthy locus, A01-2181079, was repeatedly associated with LW, WFS, DFS, and WPP and overlapped with the InDel-GWAS signal (*P =* 5.99 × 10^−7^, Fig. 5, Table 1, and Supplementary Tables S9 and S10). The corresponding gene, *Bra_cxA01g042490*, encodes DEFECTIVE EMBRYO AND MERISTEMS 1 (*DEM1*), involved in regulating plant cell division and exhibited high expression levels during stalk and leaves growth [36]. An InDel insertion in *BraDEM1* resulted in premature termination, showing a strong correlation with both traits and expression levels (*P <* 0.05), and may have been selected during the breeding process (Fig. 5c, Supplementary Fig. S13d). Additionally, the loci A02-1569970 and A07-24297027, exhibiting characteristics similar to the previous locus, were also identified (Table 1, Supplementary Fig. S14 and Supplementary Tables S9–S11). The candidate genes *Bra_cxA02g046520* and *Bra_cxA07g009110* are annotated as Probable LRR receptor-like serine/threonine-protein kinase and PLASMODESMATA CALLOSE-BINDING PROTEIN 4 (*PDCB4*), respectively. Interestingly, *PDCB4* is homologous to the candidate gene *PDCB1*, emphasizing the genetic connectivity influencing these phenotypes.

### Improved loci for yield-related traits in modern breeding

**Figure 6:**
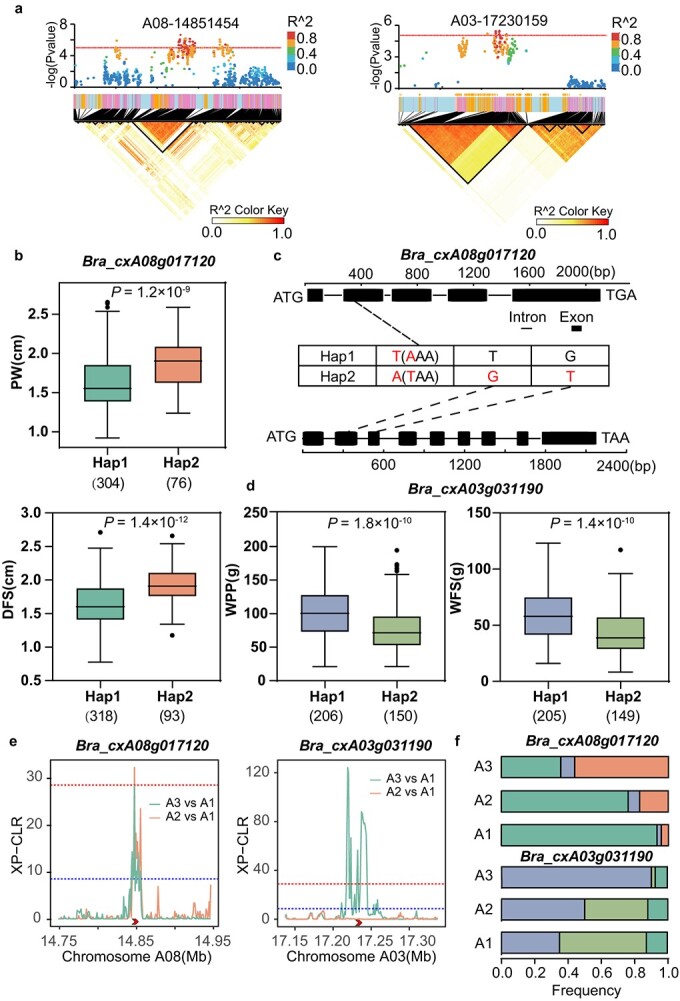
Identification of candidate genes *Bra_cxA08g017120* and *Bra_cxA03g031190* from the Intersection of GWAS and yield improvement signals. (a) Local Manhattan plot (top), gene models (middle), and LD heatmap (bottom) surrounding the A08-14851454 and A03-17230159. The horizontal dashed line represents the significance threshold (*P <* 1 × 10^−5^). (b, d) Boxplot of PW, DFS, WPP, WFS for the haplotypes (Hap) of *Bra_cxA08g017120* and *Bra_cxA03g031190*. Center line, median, box limits, upper and lower quartiles; whiskers, 1.5× the interquartile range; and dots represent outliers. Significant differences between the haplotypes were evaluated by a two-tailed *t*-test and shown by *P*-value or different letters (*P <* 0.05). (c) Exon–intron structure and DNA polymorphism of *Bra_cxA08g017120* and *Bra_cxA03g031190*. (e) XP-CLR plot of *Bra_cxA08g017120* and *Bra_cxA03g031190*. The ‘>’ symbol indicates the position of a selective region. The red and blue horizontal dashed line indicates the genome-wide cutoff of 1% and 5%, respectively. (f) Frequency changes of *Bra_cxA08g017120* and *Bra_cxA03g031190* haplotypes in different breeding periods.

A total of 113 GWAS loci, coinciding with previously recognized selection signals, were identified to pinpoint genomic loci favored by humans in modern breeding (Supplementary Table S12). Among them, 66.4% (75/113) were associated with yield-related traits. For instance, the locus A08-14851454 located on the gene *Bra_cxA08g017120* encoding an ATP-dependent DNA helicase was repeatedly associated with PW and DFS (*P =* 2.17 × 10^−13^, Fig. 6, Table 1, Supplementary Fig. S15). Another example was the locus A03-17230159 associated with WFS and WPP (*P =* 1.77 × 10^−6^). The corresponding gene *Bra_cxA03g031190* encodes a transducin/WD40 repeat-like superfamily protein, exhibiting high expression levels during stalk and leaves development and involving complex interaction networks (Supplementary Fig. S15a). Importantly, nonsynonymous mutations in these genes showed a high correlation with gene expression levels and phenotypes, and the phenotypic advantageous haplotypes may be selectively targeted and retained in breeding practices (especially during yield improvement stages A3), thereby promoting the enhancement of associated traits (Fig. 6b–f, Supplementary Fig. S13c).

### Genetic network of loci associated with agronomic traits

In crops, the most important agronomic traits are highly complex quantitative traits controlled by multiple loci [37]. This study found that 11 traits related to structure, growth, and yield showed commonly associated loci in their respective categories, indicating coregulation of genes (Supplementary Tables S9 and S10). Based on LD between GWAS loci, further network analysis suggests that these loci may be connected. Loci that control related phenotypes are more likely to be clustered together in a tight genetic network, especially for yield-related traits (Supplementary Fig. S16). In Brassica crops, leaves and enlarged organs (such as roots, stalks, and inflorescences) are important morphological features determining their economic value [38]. This study identified 73 pleiotropic QTL that simultaneously regulated traits related to stalks, leaves, and yield (Supplementary Tables S13). For instance, the association signals A09-1668557, A01-2181079, A08-14851454, and more exhibited pleiotropic associations with DFS, WFS, LW, PW, and WPP. The results indicate the presence of a complex regulatory network among these phenotypes. Therefore, it is crucial to take into account multiple related components of the target when breeding for improvement.

## Discussion

In this study, we conducted large-scale resequencing of flowering Chinese cabbage diversity populations, resulting in the generation of a comprehensive flowering Chinese cabbage genome variation dataset. This valuable resource provides insights into the improvement process of flowering Chinese cabbage in the modern breeding. The breeding improvement of flowering Chinese cabbage can be divided into two key stages: adaptation to local environments and yield improvement. As a typical leafy vegetable, flowering Chinese cabbage has a high demand for nitrogen fertilizers, especially in the product organs, and, therefore, its growth needs to adapt to environmental changes [39]. Correspondingly, the results showed that multiple genes related to nitrate uptake and transport were selected to adapt to changes in the planting environment, such as genes *NLP7*, *CLC-a*, and *NPF7.2* [27, 28]. Similarly, genes such as *WRKY53* and *MSL10*, related to environmental response and signal transduction like oxidative stress and leaf senescence, were also selected in adaptive improvement. Additionally, stalks and leaves, as the main productive organs of flowering Chinese cabbage, genes related to the morphogenesis of the relevant plant organs, such as *RBR1, CUC2,* and *DCAF1*, were selected to be involved in yield improvement. Previous studies have shown that these genes play critical roles in the process of cell division and proliferation in Arabidopsis, thereby influencing the development and morphogenesis of various tissues and organs, including shoot apical meristem (SAM), embryo, leaf, shoot, and flower [29–32]. Moreover, the haplotypes consisting of nonsynonymous mutations in these genes have exhibited significant changes in different breeding periods.

Vernalization and flowering time are important environmental adaptation factors, so they have been under selection during crop domestication, especially in *Brassica*. Previous studies have shown that the selection of flowering-related genes in modern breeding plays a crucial role in tuber formation in turnip and ecotype improvement in Chinese cabbage and rapeseed [14, 40, 41]. This highlights the central role of these genes in the development of plant morphology and in the process of germplasm improvement for adaptation to environmental changes. Similarly, the results of this study also showed that genes related to environmental adaptation, especially to ‘leaf senescence’, ‘short photoperiod’, and ‘vernalization response’, may have undergone multiple rounds of selection in modern breeding of flowering Chinese cabbage. Correspondingly, the haplotypes of related genes showed significant changes in both breeding improvement stages, such as flowering-related genes *BRN2, PCL1*, and *VIL1*. In addition, our study identified 386 genes related to plant stem development by integrating transcriptome data, which were selected for yield improvement in modern breeding. These genes, such as *HEC1, SRS5*, and *CUC2*, are involved in various aspects including plant metabolic processes, cell structure formation, hormonal pathways, and morphogenesis [29, 30, 42–44]. The previous studies further indicate the accuracy of the above results. Overexpression of SRS genes in Arabidopsis and chrysanthemum reduced plant stem height through the hormone pathway [45, 46]. The integration of multi-omics data provides valuable insights into the molecular basis of plant breeding improvement.

Exploring the genetic loci involved in the control of target traits can promote the genetic improvement of flowering Chinese cabbage through marker-assisted breeding or genetic manipulation. Some recent reports based on genome resequencing revealed loci associated with agronomic traits of *Brassica* plants such as rapeseed and Chinese cabbage [7, 14]. However, the genetic structure of agronomic traits in flowering Chinese cabbage has not been studied in depth using large-scale germplasm, which hinders the analysis of molecular mechanisms. In our study, a comprehensive GWAS analysis of 11 agronomic traits of flowering Chinese cabbage was performed for the first time to determine the causal loci that control important traits. As a strategy for identifying candidate genes for agronomic traits, InDel-GWAS can not only verify related loci but also supplement SNP-GWAS [7, 9]. Finally, we identified 642 loci and 2608 candidate genes associated with 11 agronomic traits of flowering Chinese cabbage, providing new insights into the genetic basis of this crop.

The morphology of plants is crucial for growth and management. This study identified a significant association between the nonsynonymous SNP A02-30595775 on the *BraPDCB1* gene and both PH and LFS. The protein encoded by this gene is located in the plasmodesmata, which contains an X8 domain and can specifically bind to callose. In *Arabidopsis*, overexpressing *PDCB1* leads to higher callose accumulation and reduced movement of green fluorescent protein, indicating a potential to mediate plant intercellular communication and regulate intercellular transport [47]. In addition, the candidate gene with the signal most significantly associated with PH is *BraMKK3*, which is involved in the MAPK cascade and plant hormone signal transduction. The MAPK cascade is currently known to play a very important role in the normal growth and development of plants, including PH [48–50]. For leaf-related traits, SNP A03-28137709 was repeatedly associated with LL and PL. The candidate gene *TIE1* is known to regulate leaf size and morphology by inhibiting ‘TCP’ activity during early leaf development in *Arabidopsis* [35]. These genes also exhibited high expression levels during the stalk or leaf growth, such as the bolting stage. The candidate genes and loci mentioned above offer a valuable reference for improving the plant architecture of flowering Chinese cabbage.

The yield of flowering Chinese cabbage, a complex trait, is directly or indirectly influenced by stalk and leaf-related traits. Phenotypic analysis results revealed that traits related to flowering Chinese cabbage could be categorized into four groups. The study identified multiple signals related to several traits within the same group, such as multiple loci on the *BraSRF3* (*Bra_cxA09g068050*) gene being repeatedly associated with four yield-related traits. *BraSRF3* encodes a leucine-rich repeat LRR-RLK, with its ligand STRUBBELIG (SUB) playing a role in cell morphogenesis in various organs like stalks and leaves, including control over cell division plane direction, cell number, size, and shape [51, 52]. Research has indicated that *SRF* genes in *Arabidopsis* may impact various aspects of cell wall biology. Specifically, the homologous gene *SRF4* has been demonstrated to directly promote an increase in leaf size, while *SRF3* plays a crucial role in coordinating root growth, iron homeostasis, and immune pathways through callose synthase regulation, suggesting *SRF3*'s potential impact on related plant phenotypes [53]. Additionally, previous studies have also mapped multiple correlated traits of flowering Chinese cabbage to the same chromosomal interval, further supporting our research findings [54]. Similarly, SNP (A01-2181079) was also associated with four yield traits. The key candidate gene *BraDEM1* (*Bra_cxA01g042490*) plays a vital role in plant cell division and is required for shoot tip formation and seedling development [36]. Homozygous *dem1* mutants exhibit a loss of the shoot apical meristem in tomatoes and are likely to achieve it through interaction with RAN1, a highly conserved protein that controls cell division in all eukaryotes [55, 56]. These pieces of evidence suggest that premature termination of *BraDEM1* may affect cell division, leading to phenotypic changes.

Combining selection signals with GWAS results can further explore the genes controlling key traits applied in breeding. The results indicate that genes *Bra_cxA08g017120* (ATP-dependent DNA helicase) and *Bra_cxA03g031190* (a transducin/WD40 repeat–like superfamily protein) are identified to be associated with multiple traits and show strong selection signals in their corresponding genomic regions. Previous research indicates that the former gene can regulate plant height in wheat, while the latter gene's interactions have significant effects on various aspects of growth and development in *Arabidopsis*, particularly in regulating SAM and flowering [57–61]. Consequently, these findings indicate the potential importance of these genes in the manifestation of related traits. More importantly, the phenotypic advantage haplotypes of the above genes gradually increased in the breeding improvement stage, indicating that they have been targeted for application in modern breeding practice. Apart from genes mentioned above, we also identified other key candidate genes, including *BraPDCB4*, probable LRR receptor-like serine/threonine-protein kinase, and so on. Direct or indirect evidence suggests that these genes may impact related traits [47, 62]. The repeated correlation based on traits and methods implies the reliability of these results and highlights the importance of the related candidate genes and loci in the regulation of agronomic traits of flowering Chinese cabbage.

In general, our study revealed the genomic variation and artificial selection or adaptation of different germplasms during flowering Chinese cabbage breeding. These findings offer valuable resources to elucidate the genetic basis of agronomic traits and advance future crop improvement breeding efforts.

## Methods

### Sampling and sequencing

The 403 accessions of flowering Chinese cabbage used in this study were highly pure and self-pollinated lines that were derived from multiple generations of self-fertilization and purified from germplasm collected from various regions across China by the Guangzhou Institute of Agriculture Science in Guangzhou, Guangdong, China. These accessions, to some extent, represent the biological and phenotypic diversity of flowering Chinese cabbage germplasm in China. Young leaf tissues were collected for each accession and immediately frozen in liquid nitrogen until DNA extraction and sequencing. DNA libraries were prepared according to the manufacturer's standard protocols at Novogene (Beijing, China) (https://cn.novogene.com) and sequenced on a NovaSeq 6000 platform, generating paired-end reads with a length of 150 bp.

### Planting and phenotyping

All materials in this study were planted in Nansha Science Base of the Guangzhou Institute of Agricultural Science (22.70°N, 113.56°E) during the period from October to December each year. All materials were subjected to the same field management measures, with 50 materials planted in a fixed row length interval. The experiment employed a completely randomized block design, which was repeated three times. Phenotypic identification of the flowering Chinese cabbage population was conducted over three consecutive years (2019–21). Data collection involved the selection of more than three plants with consistent growth at maturity for each material. At maturity, the flowering stalk is level with the top of the uppermost leaves, and the floral buds are just beginning to open. A total of 11 agronomic traits related to morphological characteristics and yield composition were statistically analyzed. All measurements were performed according to the standard of the Chinese Crop Germplasm Resources Information System (https://www.cgris.net/). To minimize the impact of errors on the results, abnormal values in the phenotypic data were eliminated using the 3σ criterion. The R packages lme4 (v.1.1.31) and lsmeans (v.2.30.0) were utilized to calculate the BLUE value and generalized heritability of the traits [63, 64]. The *H*^2^ was estimated under the following formula: *H*^2^ = δ2_g_/(δ2_g_ + δ2_ge_ + δ2/r). In this equation, δ2_g_ represents the genetic variance, δ2_ge_ represents the interaction variance of genotype × environment, δ2 represents the error variance, and *r* represents the replications. Descriptive statistics and Pearson correlation coefficients between the 11 phenotypic data were computed using the R package psych (ver.2.2.9, https://CRAN.R-project.org/package=psych), and visualizations were generated [65]. Subsequently, word hierarchical clustering was performed on the phenotypic data using the R package factoextra (ver.1.0.7, https://cloud.r-project.org/package=factoextra/), enabling an analysis of the phenotypic characteristics of the flowering Chinese cabbage population.

### Reads alignment and variation detection

A total of 1279.13 G raw data was obtained in this study through the whole-genome resequencing of 403 flowering Chinese cabbage materials. To eliminate any potential artificial bias, strict standards were applied to remove low-quality data and connectors. These standards included: (i) determining that the number of uncertain nucleotides in any sequencing read exceeded 10% of the total number of bases; (ii) ensuring that the number of low-quality bases (*Q* ≤ 5) in any sequencing read exceeded 50%; (iii) confirming the absence of adapter sequences in the sequencing read; and (iv) identifying and removing potential duplications resulting from Polymerase Chain Reaction (PCR) amplification during library construction. As a result of these criteria, 1245.42G clean data was obtained. Additionally, FastQC (ver.0.12.1) was employed to create sequencing data quality reports [66].

To align clean paired-end reads to the Youlv 701 reference genome and produce bam files, we employed BWA-MEM (v0.7.17) [67]. Following this, we utilized the Samtools software (ver.1.15) to convert and index the alignment results into bam files [68]. To enhance the alignment results, potential PCR repeats were eliminated. Subsequently, we jointly invoked the SNP and indel of the flowering Chinese cabbage population using the default parameters of GATK HaplotypeCaller and VariantFiltration (ver.4.2.6.1), following the recommended hard filtering method [69]. The variation data was filtered using parameters (-maf 0.05, -biallelic-only, and -gene 0.20) through the plink software (ver.1.90) [70]. In the subsequent analysis, any missing data were filled in using the Beagle software (ver.5.4) with default parameters [71]. Finally, the variations were annotated using the Snpeff software (ver.5.1) [72].

### Population genetic and linkage disequilibrium analysis

To investigate the structure and phylogenetic relationship of the flowering Chinese cabbage population, various tools were employed. Firstly, the filtered SNPs were used to construct an individual-based NJ tree using MEGA7 [73]. The resulting tree was visualized in iTOL (itol.embl.de/). Plink (v.1.90) was utilized to calculate feature vectors and eigenvalues, while the R language facilitated visualization. The association analysis incorporated significant principal components. The Normalized_IBS model of Tassel (ver.5.2.40) was used to calculate the genetic correlation between individuals, and this correlation was visualized using the R-package pheatmap (version.1.0.12) [74]. ADMIXTURE (ver.1.30, -C 0.01 -j24) was applied to construct the genetic structure and pedigree information of the population, which was subsequently visualized by Tbtools (v.1.123) [75, 76]. Additionally, PopLDdecay (ver.3.41) software was used to evaluate the LD decay of the population, with a maximum distance of 500 kb [77]. This software directly utilized the VCF format file for input and called the pipeline for visualization. Nucleotide diversity (π) was estimated to quantify the degree of variation within each subgroup, while fixation index (Fst) was employed to explain genetic differentiation. VCFtools (v.0.1.16) was used to calculate both π and Fst [78]. Finally, six turnip samples (TUE and TUA, three samples each) closely aligned with ancestral relationships were selected as outgroup for constructing the phylogenetic tree using MEGA7 and the p-distance method.

### Identification of selective sweep signals

To analyze the selected regions during different breeding periods of flowering Chinese cabbage, we used VCFtools (ver.0.1.16) to calculate fixation statistics (Fst) and nucleotide diversity (π). For this analysis, a sliding window approach with a window size of 20Kb and a step size of 10Kb was employed. To convert the distribution of Fst and π ratio, we applied the logarithm function. Windows that had log-odds ratios in the top 5% for both Fst and π were considered as the putative selection target regions. Additionally, we utilized the XP-CLR program in the XP-LCR method (v.1.1.2), assessing each chromosome individually with the parameters: '-w1 0.0005 200 200 1 -p1 0.9′ [79]. To calculate average XP-CLR scores, we used nonoverlapping 10Kb sliding windows with a step size of 1Kb. We then categorized these windows into separate features. The top 1% window of XP-CLR score was identified as a candidate selective region, and downstream analysis focused on the genes within this region. The gene set was subjected to GO enrichment analysis using the clusterProfiler (v.4.4.4) R package, applying a *P <* 0.05 filtering threshold. Redundancy in the enrichment results was subsequently eliminated using REVIGO (v.1.8.1) [80].

### Genome-wide association analysis of agronomic traits

We performed a large-scale association analysis of 11 traits in 403 flowering Chinese cabbage materials, based on 2 515 078 high-quality SNPs and 656 098 indels (maf > 0.05, miss <0.2). To conduct this analysis, we utilized the Genome-wide Efficient Mixed Model Association (GEMMA) software (v.0.98.3) to apply the linear mixed model (LMM) of kinship matrix (K) and K + principal components [81]. Additionally, we employed the Bayesian-information and Linkage-disequilibrium Iteratively Nested Keyway (BLINK) model of the R package GAPIT to conduct the association analysis [82]. To determine the GWAS analysis threshold, we used GEC (v0.2) to calculate the effective number of SNPs (Me) [83]. This allowed us to reduce the strictness of the Bonferroni correction (*P <* 1.46 × 10^−6^). However, for some less significant associated traits, this threshold remained too strict, so we used a relatively loose standard (*P <* 1 × 10^−5^) to detect SNPs associated with traits, which served as our secondary threshold. Finally, we utilized the R software (ver4.2.0) packages CMplot (v.4.4.1) and qqman (v.0.1.9) to draw the Manhattan.

By employing LDBlockShow (v.1.40, -SeleVar 2 -BlockType 1) software, we conducted LD block analysis for the associated loci to examine their local LD [84]. Our primary focus resides in the nonsynonymous SNPs within the variation sites of the linkage region. Consequently, we classified the samples into distinct haplotypes using these sites. We subsequently performed a two-tailed *t*-test to determine the disparities between the different haplotype phenotypes, thereby confirming the causal SNPs for each gene. The STRING v12.0 database (https://string-db.org) was utilized to predict the protein–protein interaction network of core candidate genes.

### Construction of association networks

To elucidate the complex regulatory network between different traits, we constructed an association network using traits and their corresponding loci. Each node in the network represents a trait or locus, and the thickness of the connection between nodes represents the degree of association. The relationship between nodes is determined by the average LD between the corresponding loci. To calculate the mean value of *r*^2^ between pairs of SNPs, we utilized Plink (v.1.9.0) software. Finally, the visualized network was constructed using Cytoscape (v.3.3.0) [85].

### Expression analysis with RNA-seq and Quantitative Real-time PCR (qRT-PCR)

Expression analysis was conducted using RNA-seq data of stalks and leaves at different stages, which were downloaded from the National Center for Biotechnology Information [33, 34]. To ensure data quality, the original data were processed using Fastp (v.0.12.4) software [86]. Subsequently, the data were compared to the ribosomal database Ribosomal Database Project using bowtie2 software (v.2.4.5) to remove rRNAs and obtain high-quality clean reads [87]. The clean reads were then aligned to the reference genome using HISAT2 software (v.2.2.1) [88]. Next, the expression levels of each transcript were counted and normalized to transcripts per kilobase of exon model per million mapped reads (TPM) values using featureCounts software (v.2.0.1) [89]. Differential expression analysis was performed using the R package DESeq2 (v.1.34.0), with a significance threshold set at *P* ≤ 0.05 and an absolute value of log2 (fold change) ≥ 1 to identify differentially expressed genes.

For each haplotype, we randomly selected five samples for qRT-PCR analysis to examine their expression changes (Supplementary Table S14). Total RNA was extracted from the stalks and leaves during the bolting stage using the Eastep® Super Total RNA Extraction Kit (Promega) and was analyzed by qRT-PCR. The extracted total RNA was reverse-transcribed using the HiScript® II Q RT SuperMix for qPCR (+gDNA wiper) kit (Vazyme), and subsequent qRT-PCR analyses were performed using the ChamQ Universal SYBR qPCR Master Mix kit (Vazyme). The gene *GAPDH* served as an internal control. Primers of the candidate genes were designed using premier 5 (Supplementary Table S14).

## Supplementary Material

Web_Material_uhae299

## Data Availability

The transcriptome and reference genome data used in this study are derived from previously published research. Specifically, the transcriptome data of stalks and leaves were obtained from the publicly accessible National Center for Biotechnology Information (NCBI) database (https://www.ncbi.nlm.nih.gov/) under the accession numbers PRJNA390062 and PRJNA778186 [33, 34]. The reference genome sequence and the raw genome resequencing data used in this study are available in CNGBdb under Bioproject number CNP0006162 [24].
