# Supplementary material for: Genomic selection and genetic architecture of agronomic traits during modern flowering Chinese cabbage breeding
Source: Hortic Res. 2024 Oct 18;12(2):uhae299. doi: 10.1093/hr/uhae299 (PMC11822411; doi:10.1093/hr/uhae299)
Supplement: Web_Material_uhae299 [file web_material_uhae299.zip › Supplementary Figure 1-16.pdf]

SUPPLEMENTARY INFORMATION

Genomic selection and genetic architecture of agronomic traits during modern flowering Chinese cabbage breeding

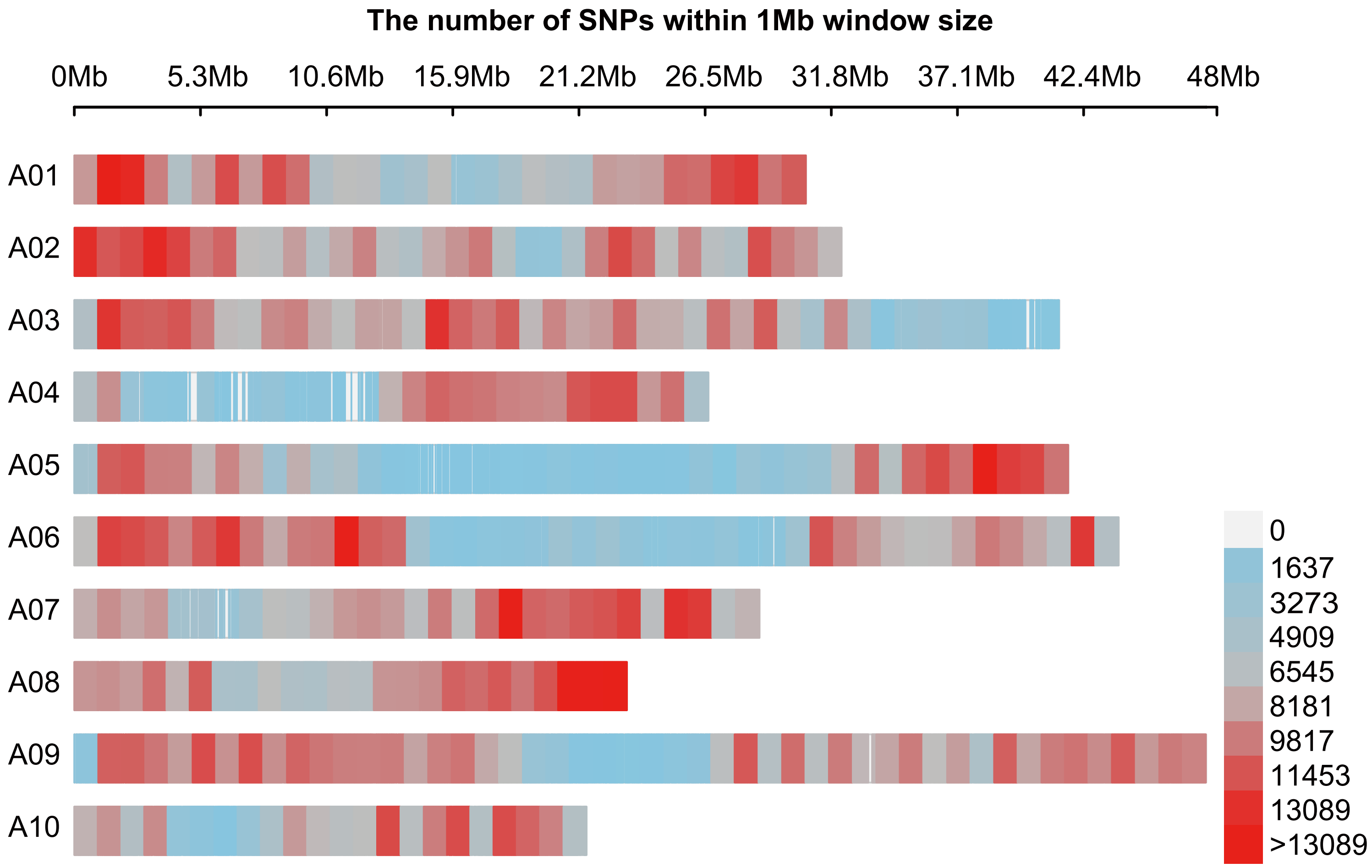

Supplementary Figure 1. SNP distribution in the flowering Chinese cabbage genome. Distribution of SNPs in the 10 chromosomes. The x-axis represents the physical distance along each chromosome, split into 1 Mb windows.

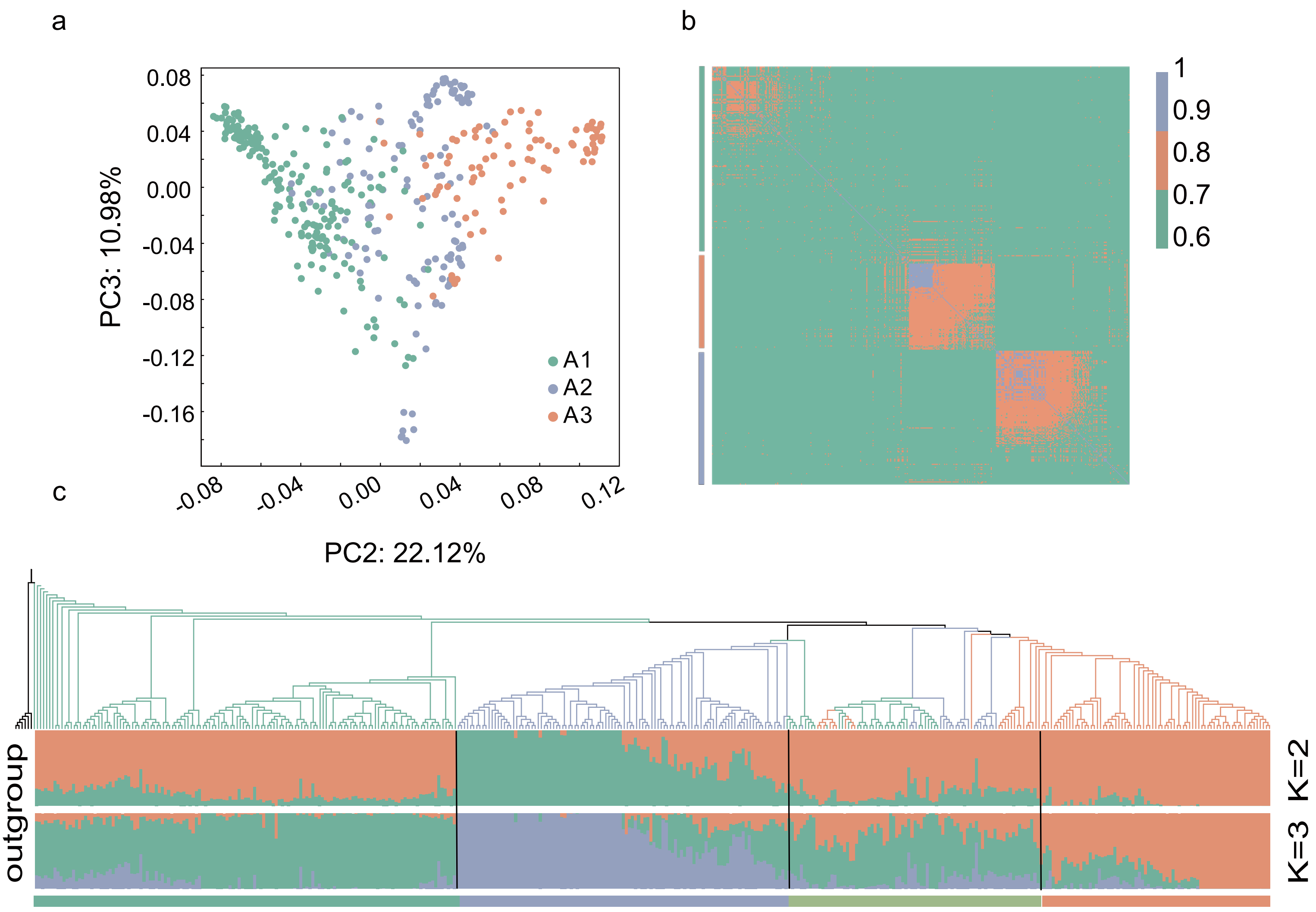

Supplementary Figure 2. Population structure and phylogenetic analysis of 403 Chinese flowering cabbage accessions. a, Principal component analysis plot of all the accessions used in this study. b, Kinship matrix based on genetic similarities (IBS, identity by state). c, Neighbor-joining tree of the 403 accessions based on the genetic distance, with Turnip as an outgroup, and Population structure for K = 2-3.

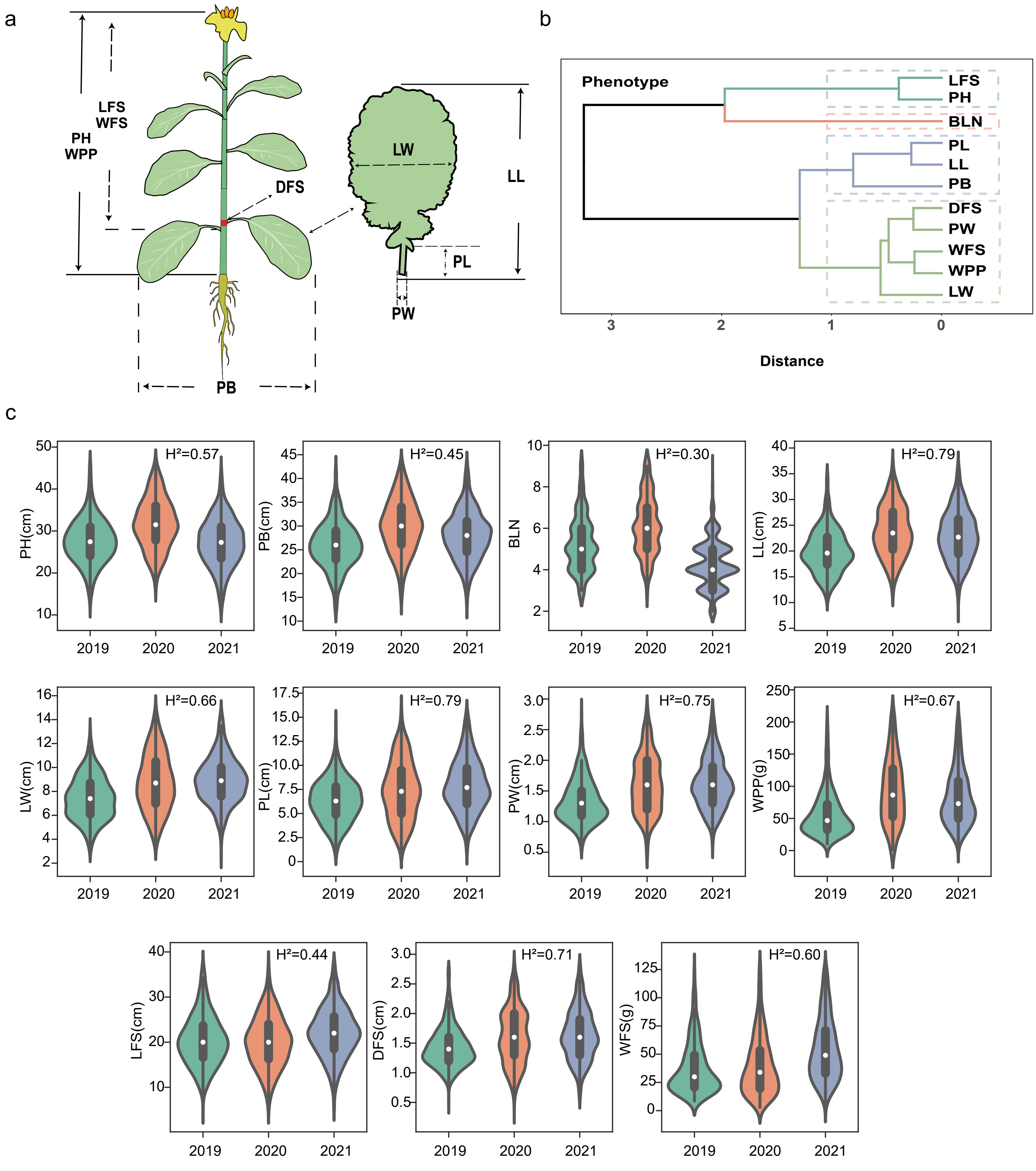

Supplementary Figure 3. Description and distribution of agronomic traits of flowering Chinese cabbage. a, Description schematic diagram of 11 key agronomic traits of flowering Chinese cabbage. b, Ward hierarchical clustering of 11 agronomic traits. c, Phenotypic distribution in three consecutive years (2019 to 2021) and broad-sense heritability (H^2) analysis.

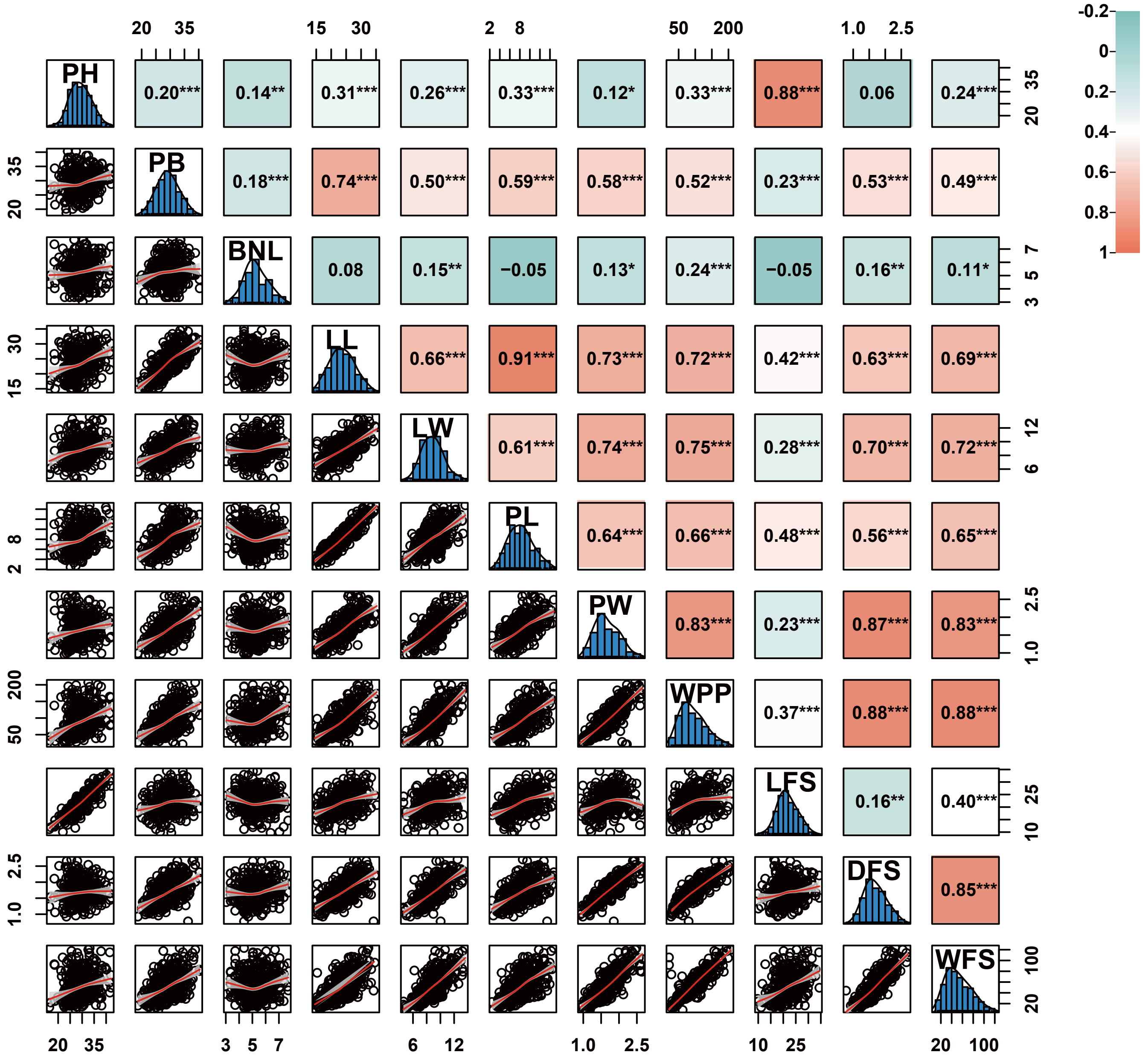

Supplementary Figure 4. Correlation coefficient among 11 agronomic traits of Chinese flowering cabbage. The pair plot shows bivariate scatter plots below the diagonal, histograms on the diagonal, and Pearson correlation between given traits above the diagonal.

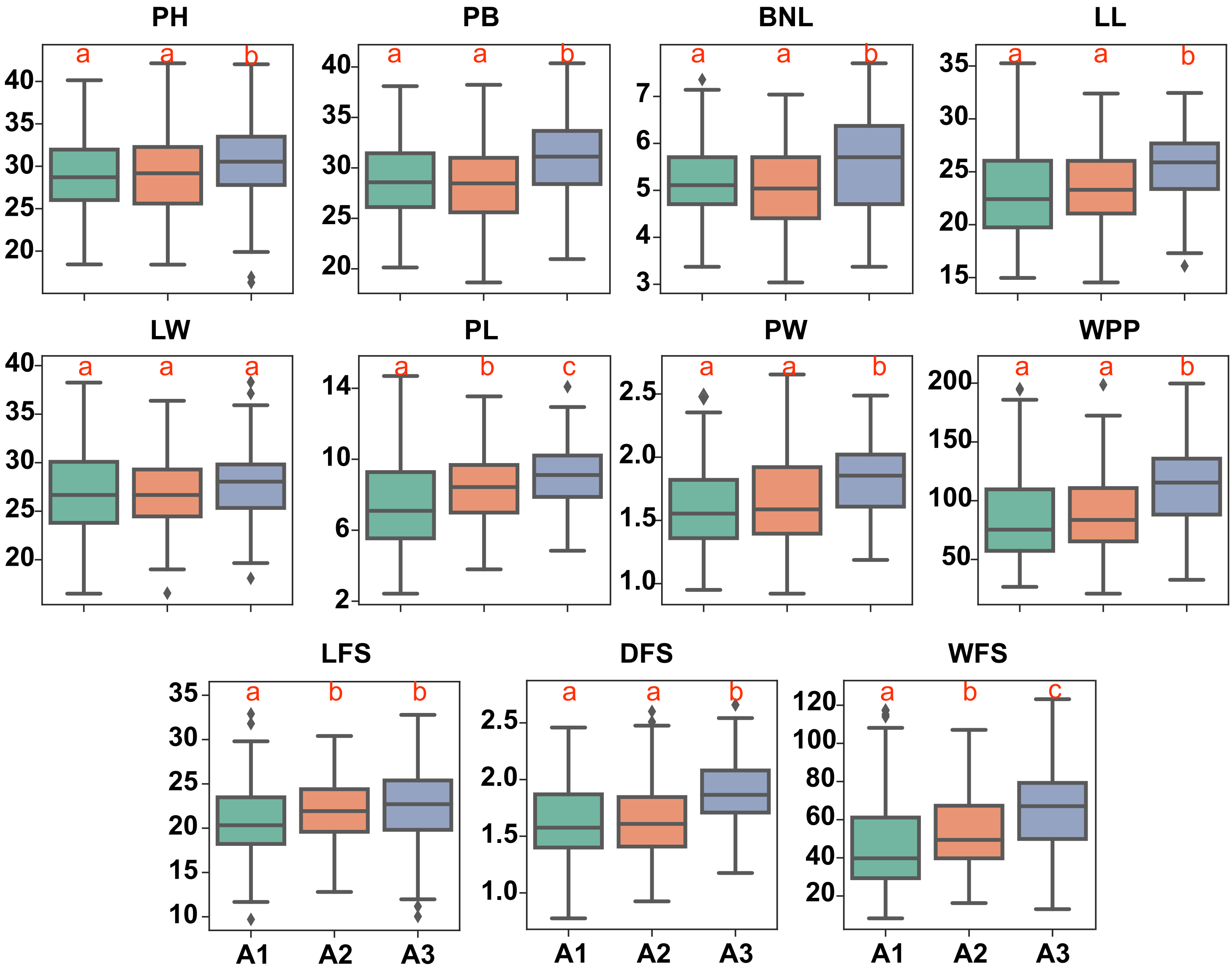

Supplementary Figure 5. Changes in agronomic traits during flowering Chinese cabbage breeding. Different letters above the boxes indicate significant differences ($P < 0.05$, two-tailed t-test). The center lines indicate the median, box limits represent the upper and lower quartiles, whiskers extend to 1.5× the interquartile range, and dots represent outliers.

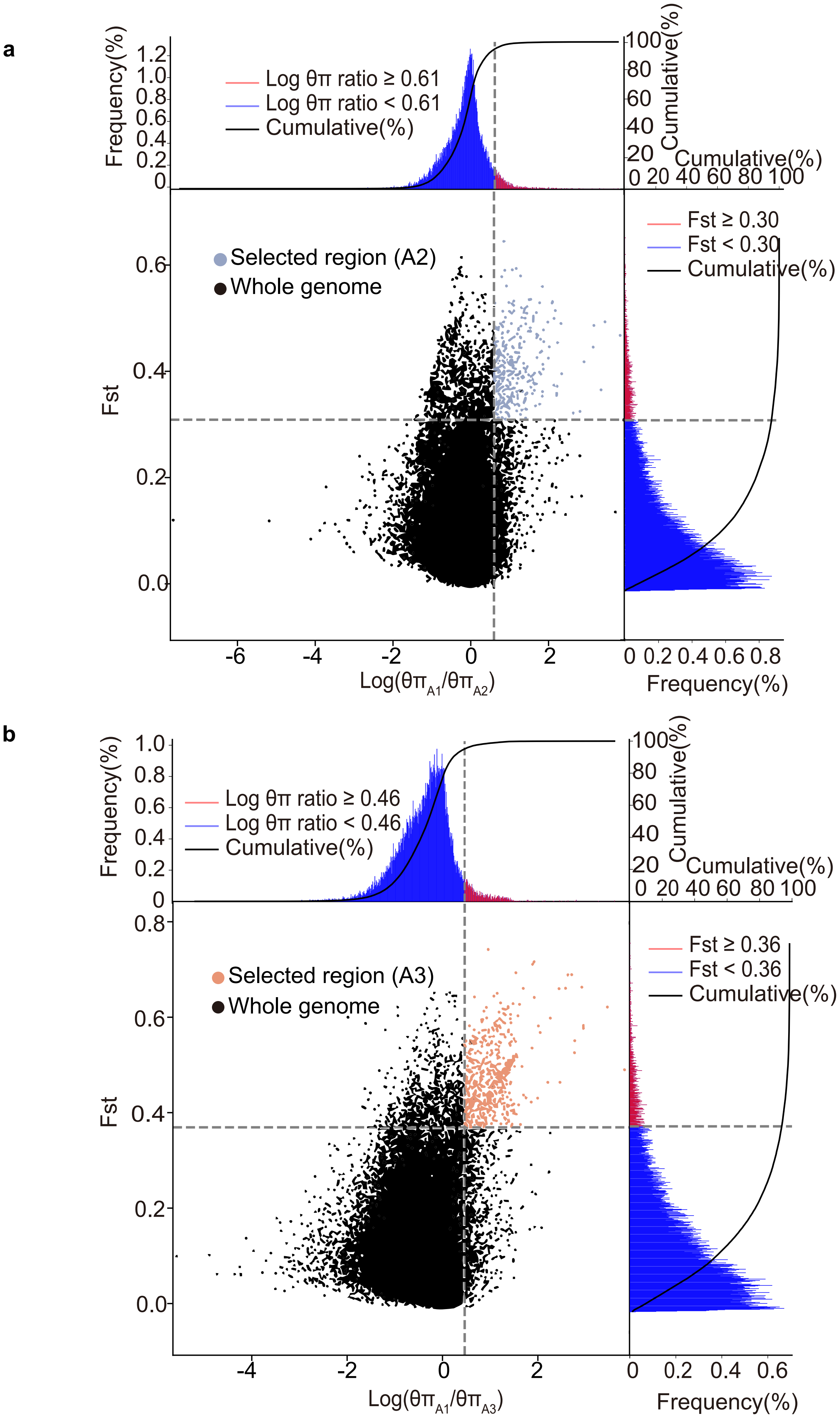

Supplementary Figure 6. Identification of improved signals in modern breeding of flowering Chinese cabbage by Fst and θπ. The abscissa is the ratio of θπ, and the ordinate is the Fst value, which corresponds to the frequency distribution map above and the frequency distribution map on the right side, respectively. The point map in the middle represents the corresponding Fst and θπ ratios in different windows. The dotted line represents the threshold of the top 5% region corresponding to Fst and θπ, and the region marked by the middle color is the intersection of Fst and θπ, which is the candidate site.

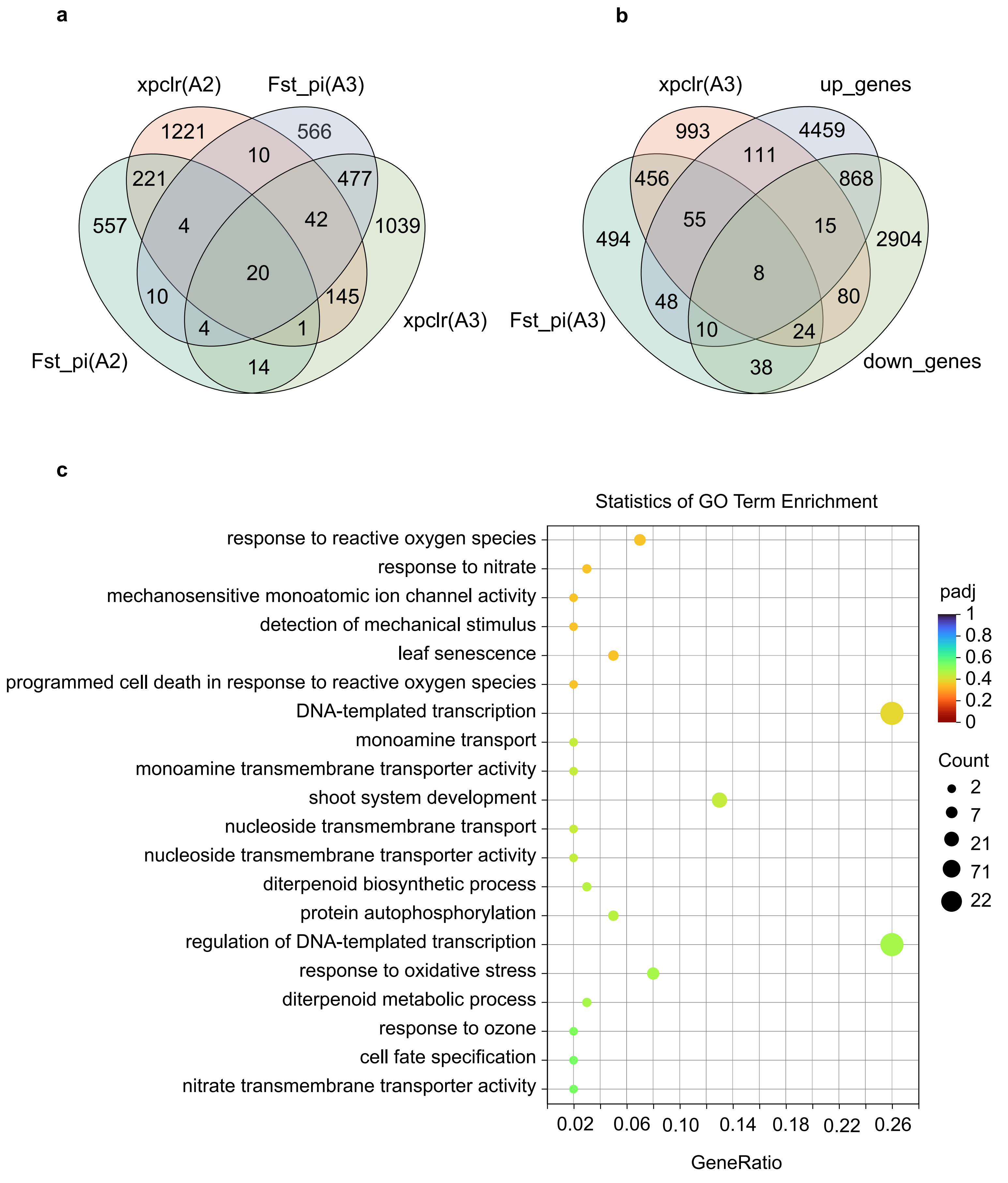

a
xpclr(A2)
Fst_pi(A3)
Fst_pi(A2)
xpclr(A3)
1221
566
10
221
477
557
4
42
1039
20
10
145
4
1
14
b
xpclr(A3)
up_genes
Fst_pi(A3)
down_genes
993
4459
111
456
868
494
55
15
2904
8
48
80
10
24
38
c
Statistics of GO Term Enrichment
response to reactive oxygen species
response to nitrate
mechanosensitive monoatomic ion channel activity
detection of mechanical stimulus
leaf senescence
programmed cell death in response to reactive oxygen species
DNA-templated transcription
monoamine transport
monoamine transmembrane transporter activity
shoot system development
nucleoside transmembrane transport
nucleoside transmembrane transporter activity
diterpenoid biosynthetic process
protein autophosphorylation
regulation of DNA-templated transcription
response to oxidative stress
diterpenoid metabolic process
response to ozone
cell fate specification
nitrate transmembrane transporter activity
0.02
0.06
0.10
0.14
0.18
0.22
0.26
GeneRatio
padj
1
0.8
0.6
0.4
0.2
0
Count
2
7
21
71
22

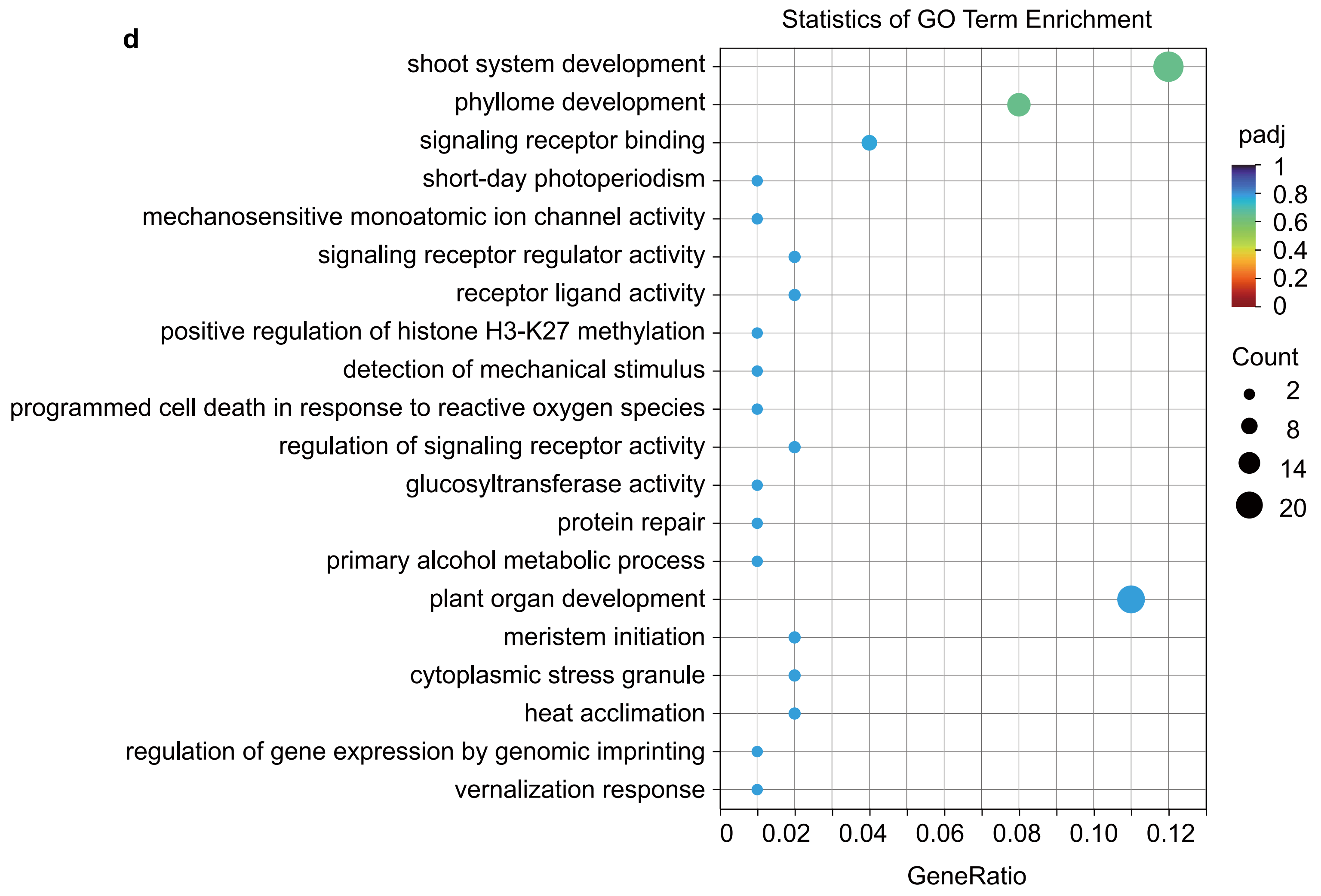
d
Statistics of GO Term Enrichment
shoot system development
phyllome development
signaling receptor binding
short-day photoperiodism
mechanosensitive monoatomic ion channel activity
signaling receptor regulator activity
receptor ligand activity
positive regulation of histone H3-K27 methylation
detection of mechanical stimulus
programmed cell death in response to reactive oxygen species
regulation of signaling receptor activity
glucosyltransferase activity
protein repair
primary alcohol metabolic process
plant organ development
meristem initiation
cytoplasmic stress granule
heat acclimation
regulation of gene expression by genomic imprinting
vernalization response
0 0.02 0.04 0.06 0.08 0.10 0.12
GeneRatio
padj
1 0.8 0.6 0.4 0.2 0
Count
2 8 14 20

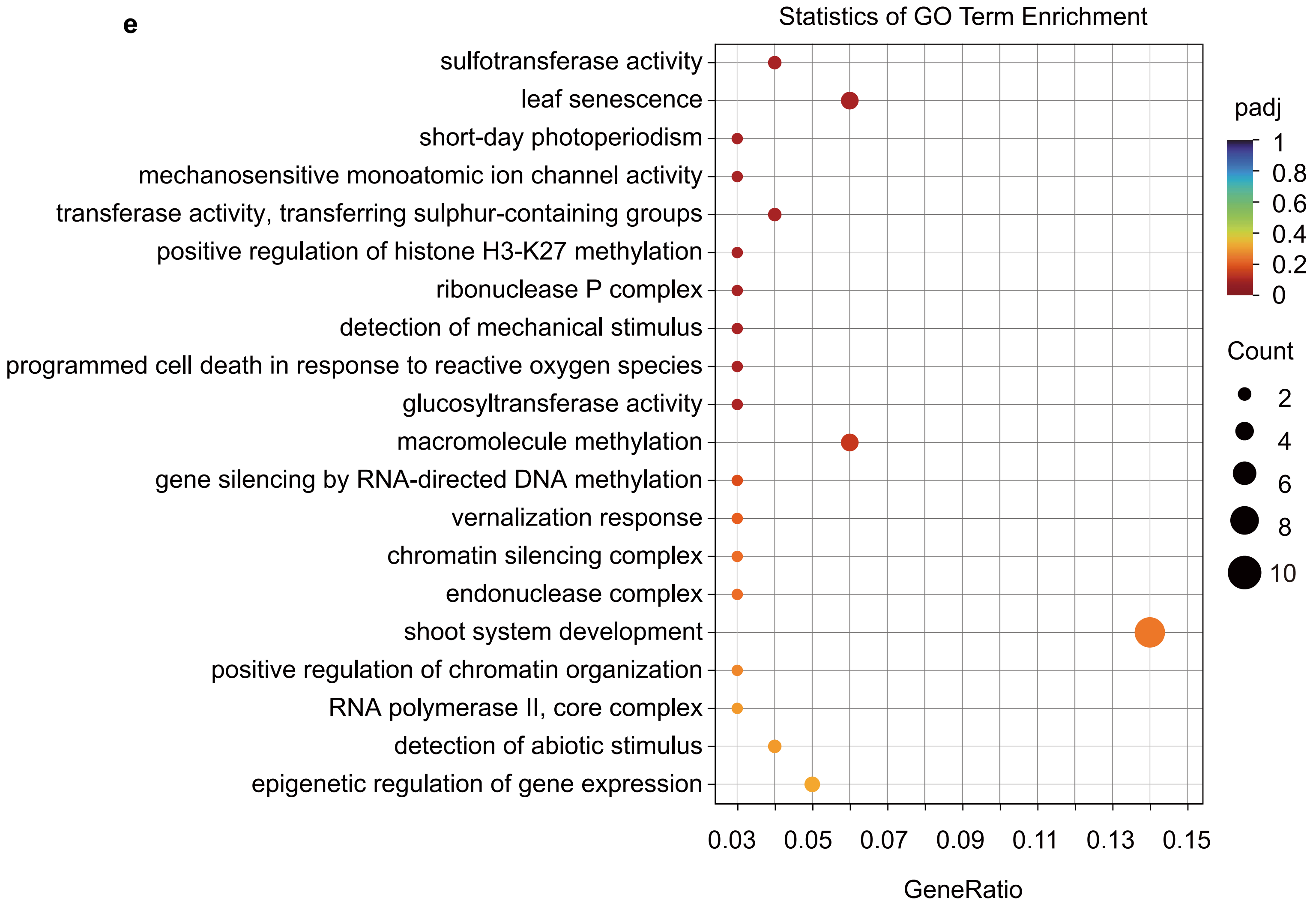
e
Statistics of GO Term Enrichment
sulfotransferase activity
leaf senescence
short-day photoperiodism
mechanosensitive monoatomic ion channel activity
transferase activity, transferring sulphur-containing groups
positive regulation of histone H3-K27 methylation
ribonuclease P complex
detection of mechanical stimulus
programmed cell death in response to reactive oxygen species
glucosyltransferase activity
macromolecule methylation
gene silencing by RNA-directed DNA methylation
vernalization response
chromatin silencing complex
endonuclease complex
shoot system development
positive regulation of chromatin organization
RNA polymerase II, core complex
detection of abiotic stimulus
epigenetic regulation of gene expression
0.03 0.05 0.07 0.09 0.11 0.13 0.15
GeneRatio
padj
1 0.8 0.6 0.4 0.2 0
Count
2 4 6 8 10

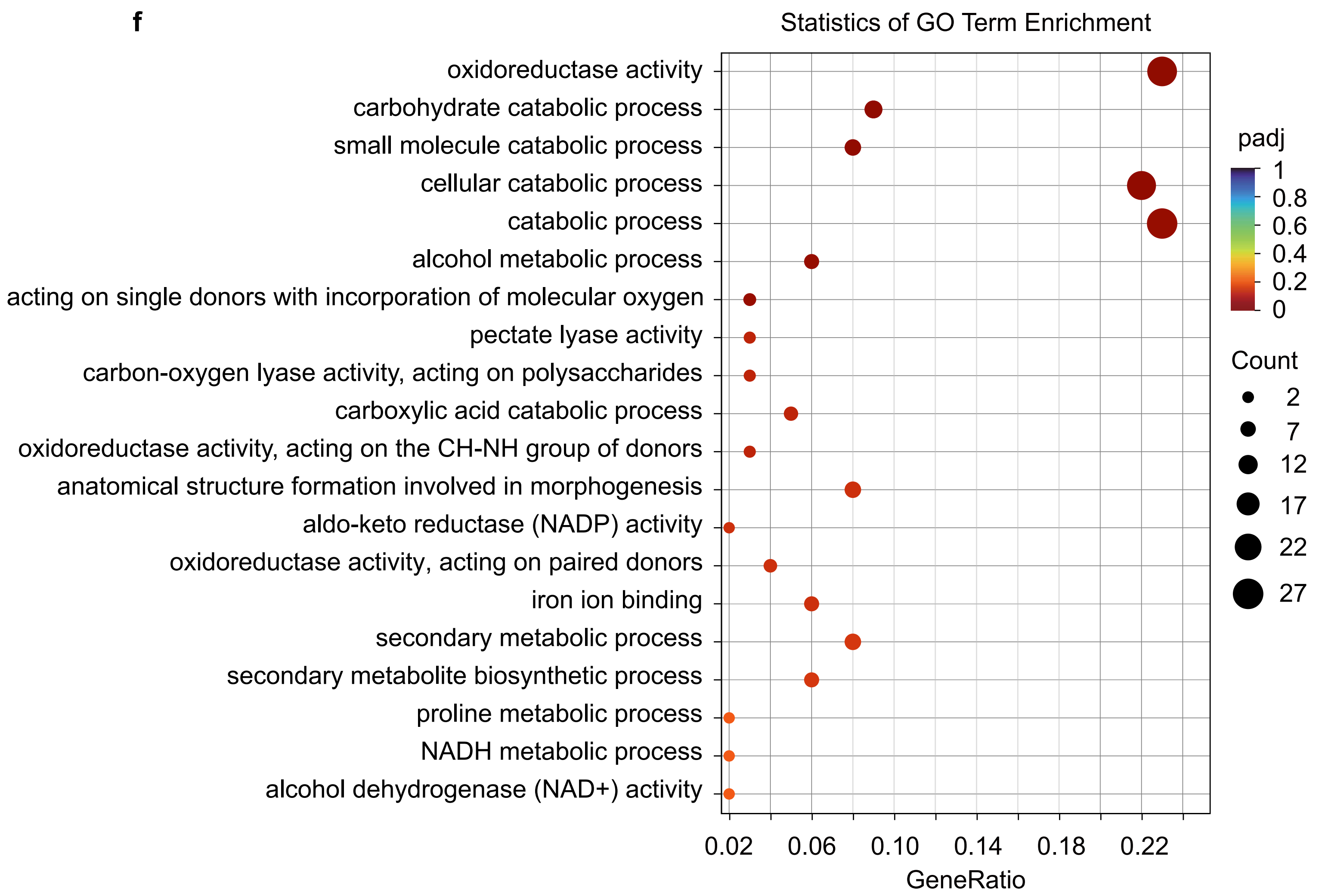

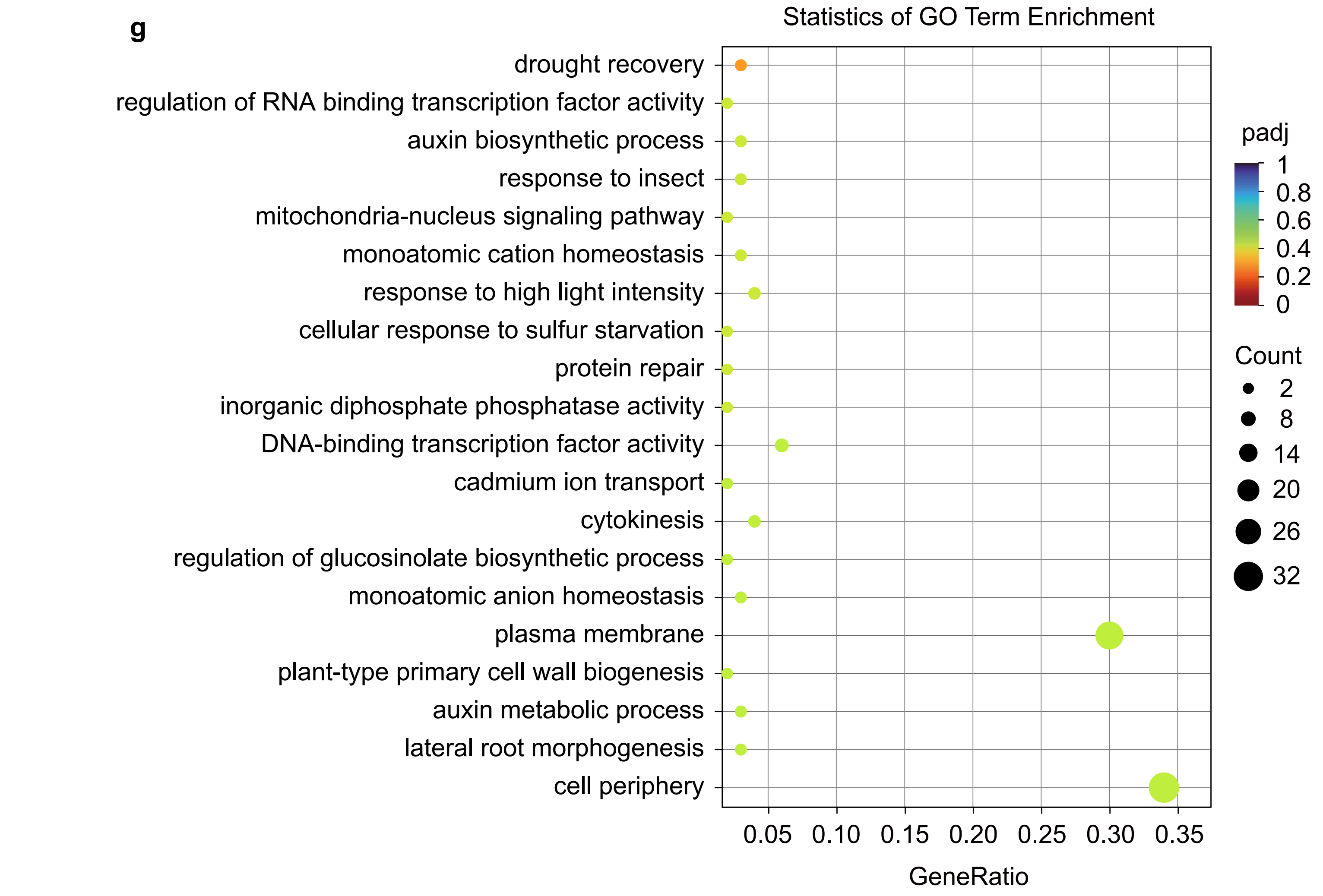

Supplementary Figure 7. GO enrichment analysis of the genes encompassed in selective sweeps. a, Number of candidate genes in the selective sweeps between different breeding periods. b, Number of intersection genes between yield improvement signal and stem development transcriptome. c-g, GO enrichment analysis of the genes encompassed in selective sweeps. c, Genes selected in adaptive improvement stage (A2). d, Genes selected in yield improvement stage (A3). e, Intersection genes of yield and adaptive improvement stage. f, The intersection of genes in the yield improvement stage and up-regulated differentially expressed genes in stem development transcriptome. g, The intersection of genes in the yield improvement stage and down-regulated differentially expressed genes in stalk development transcriptome.

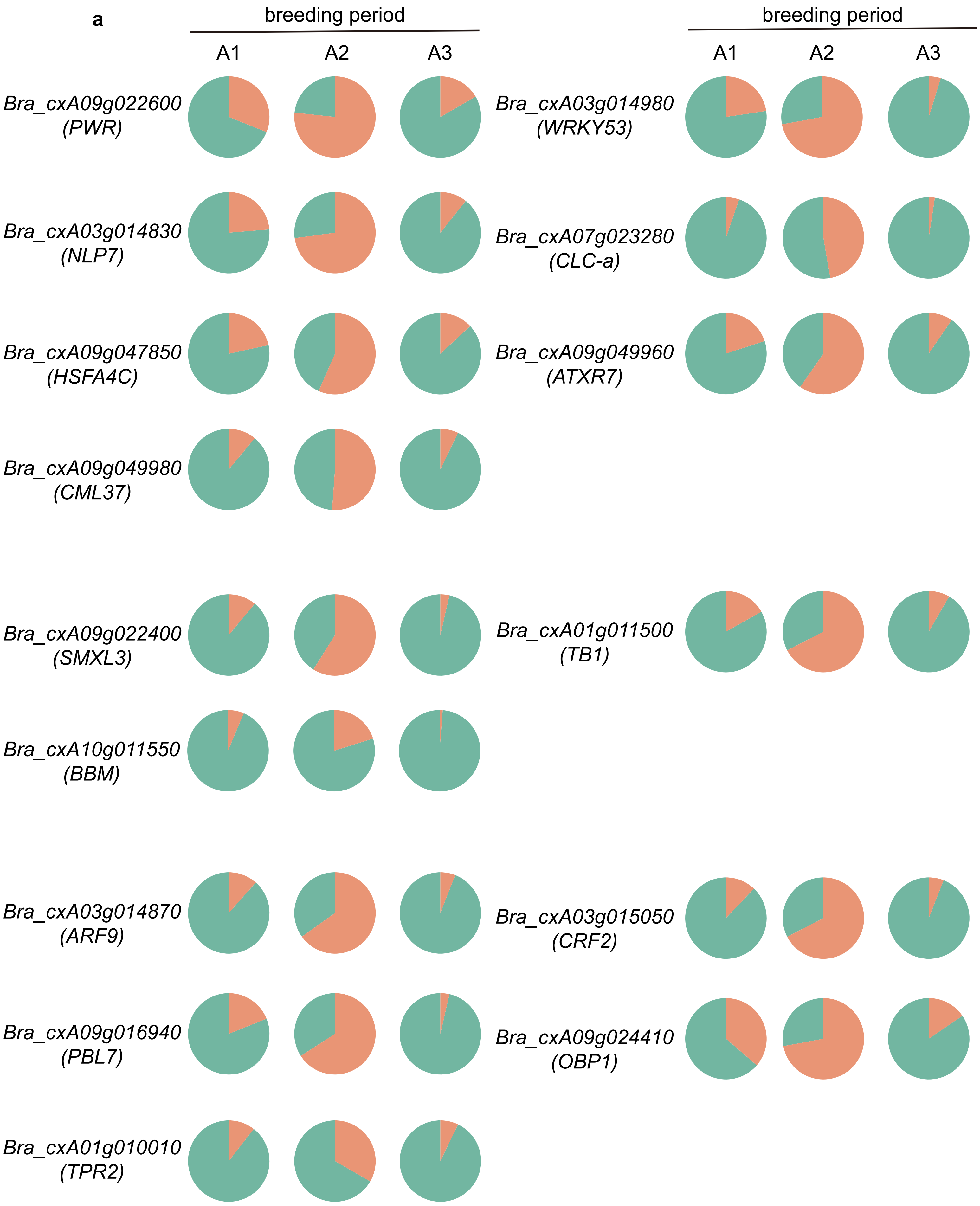

a
breeding period
A1
A2
A3
Bra_cxA09g022600
(PWR)
Bra_cxA03g014830
(NLP7)
Bra_cxA09g047850
(HSFA4C)
Bra_cxA09g049980
(CML37)
Bra_cxA09g022400
(SMXL3)
Bra_cxA10g011550
(BBM)
Bra_cxA03g014870
(ARF9)
Bra_cxA09g016940
(PBL7)
Bra_cxA01g010010
(TPR2)
breeding period
A1
A2
A3
Bra_cxA03g014980
(WRKY53)
Bra_cxA07g023280
(CLC-a)
Bra_cxA09g049960
(ATXR7)
Bra_cxA01g011500
(TB1)
Bra_cxA03g015050
(CRF2)
Bra_cxA09g024410
(OBP1)

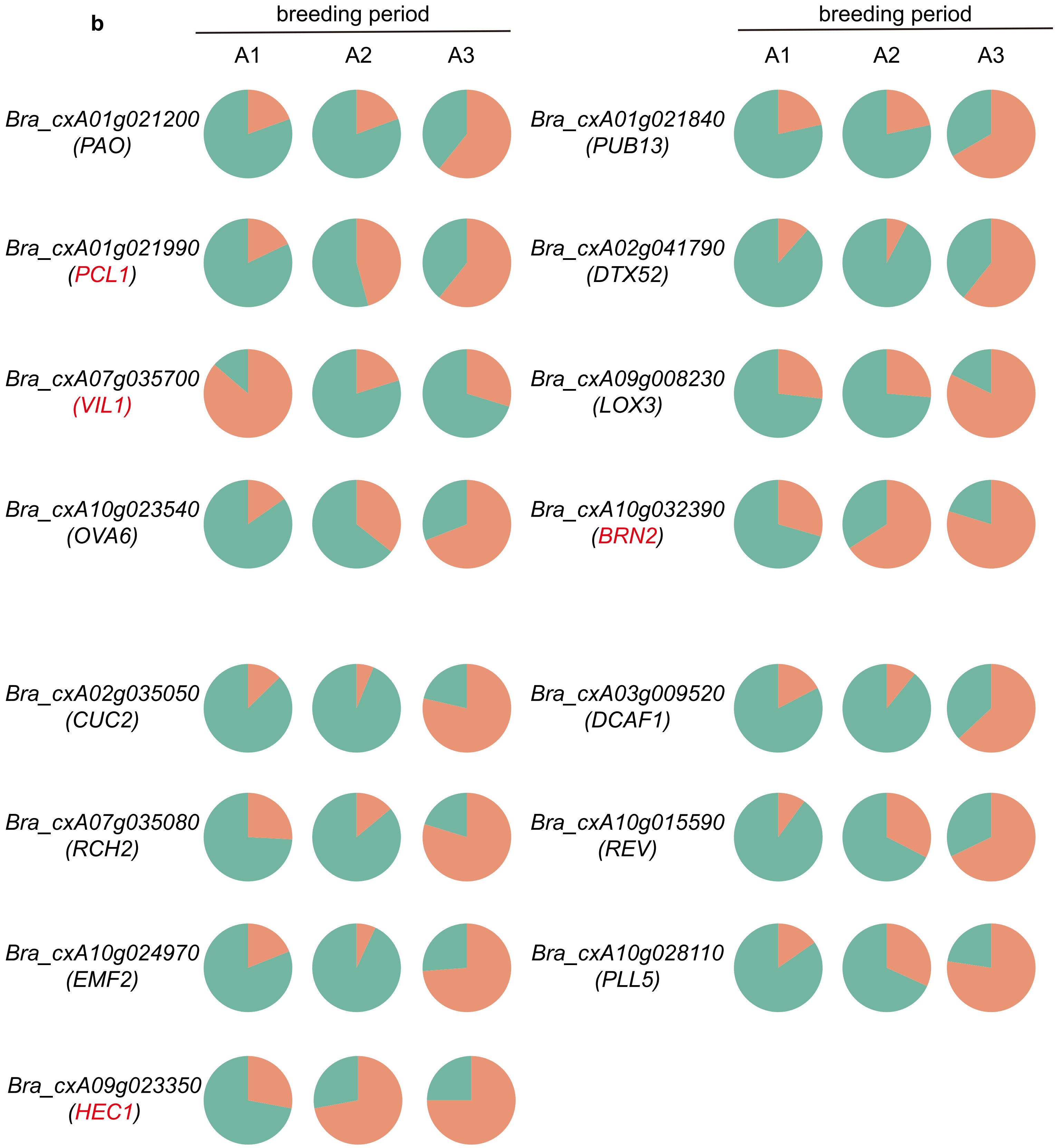

Supplementary Figure 8. Stepwise selection for agronomic traits during flowering Chinese cabbage breeding. a, In the adaptive improvement stage (A2), the allele frequency distribution of genes related to environmental adaptation, plant growth and hormone signal transduction. b, During the yield improvement stage (A3), the distribution of allele frequency of genes related to environmental adaptation, plant growth, and hormone signal transduction. Green represents homozygous haplotypes without nonsynonymous mutations, while red represents haplotype combinations that contain nonsynonymous mutations.

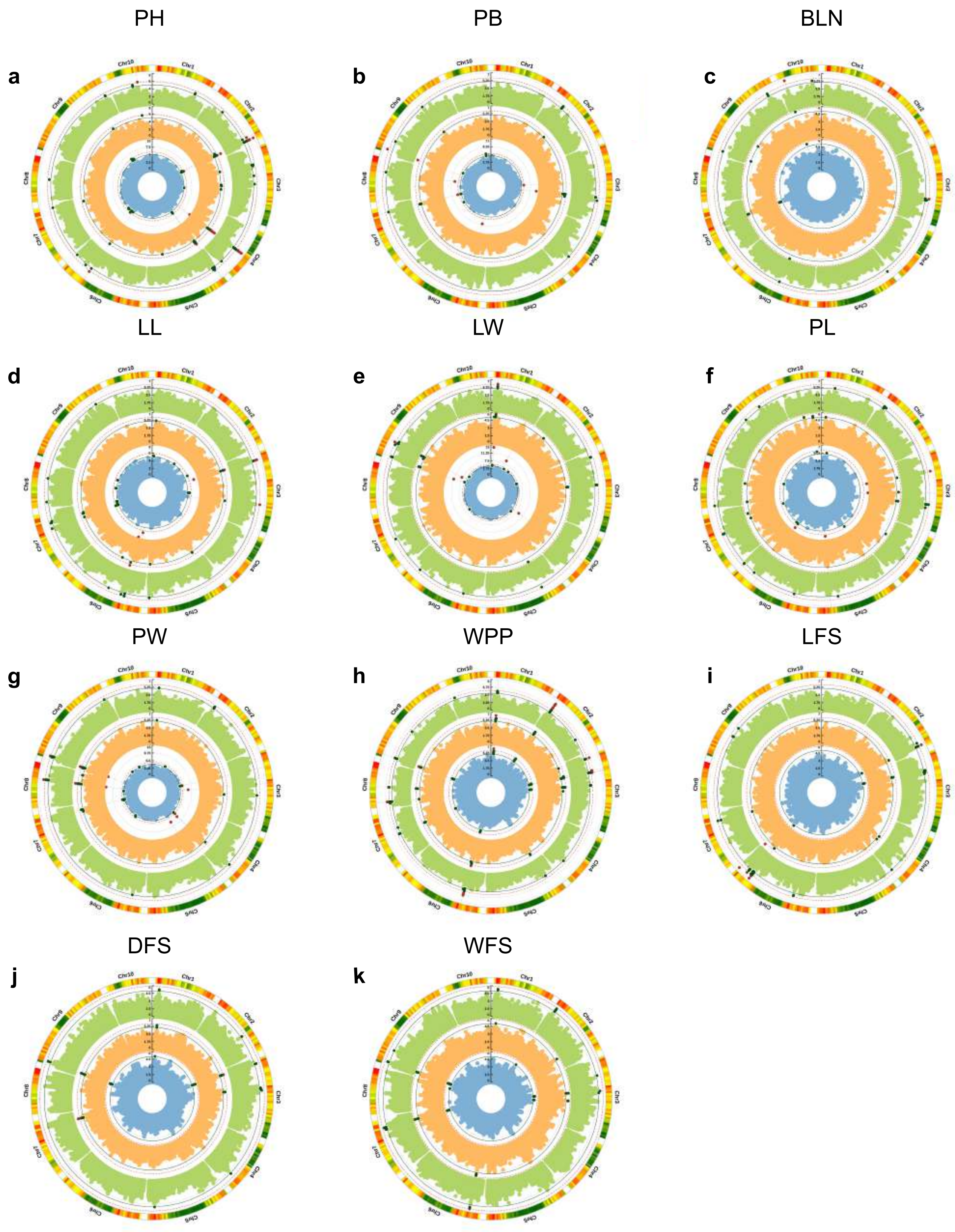

Supplementary Figure 9. Manhattan plot of SNP-GWAS for 11 agronomic traits of Chinese flowering cabbage. Each graph illustrates three methods of GWAS from the inside to the outside. These methods include the LMM model with K, the LMM model with K + P, and the Blink model. The outermost layer of each graph represents SNP density. The dark green and red points represent the points passing the second and first thresholds, respectively.

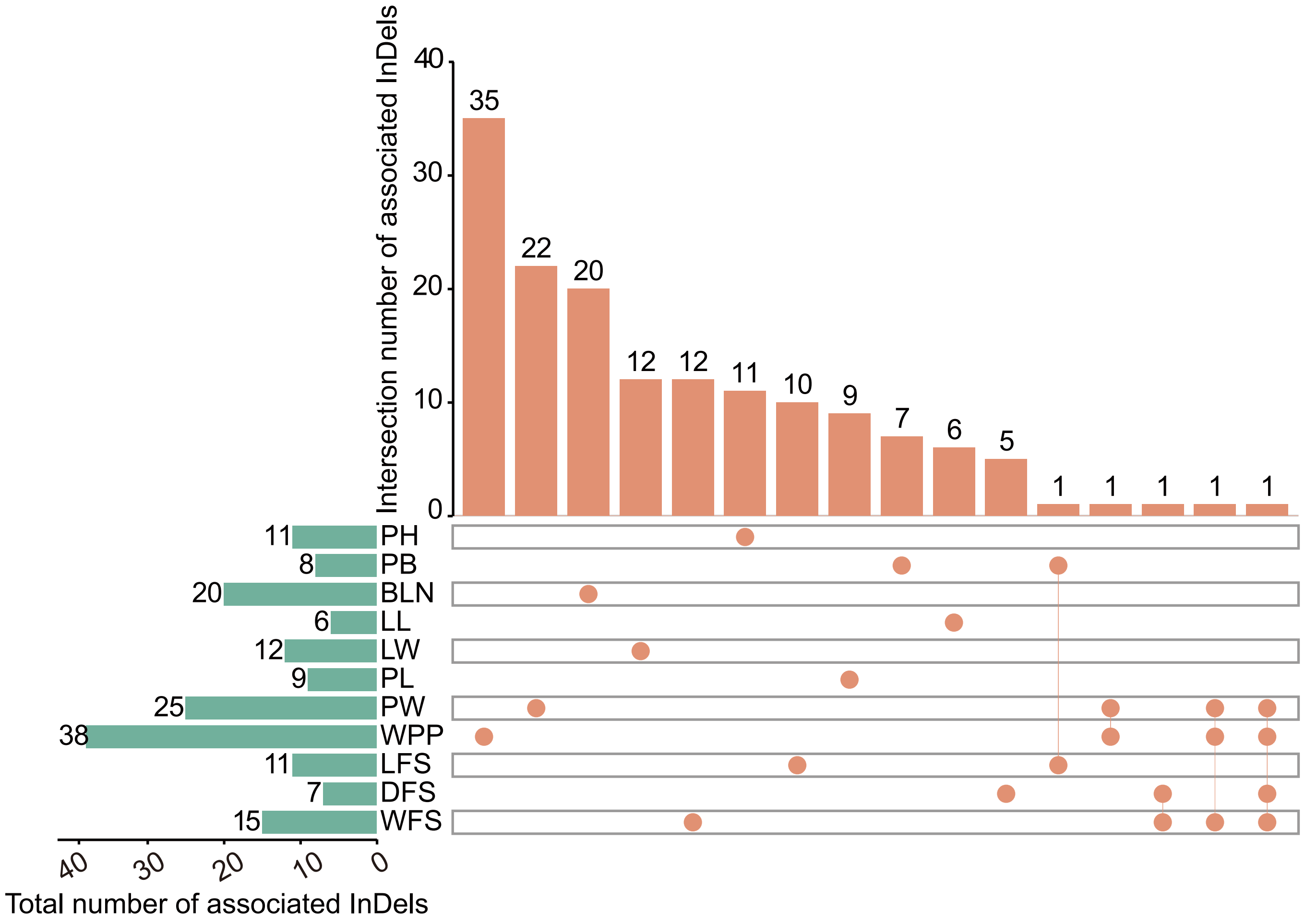

Supplementary Figure 10. Upset Venn diagram showing the overlap number of the InDels associated with the 11 agronomic traits. The horizontal histogram at the left shows the total number of InDels in each agronomic trait. The vertical histogram at the top shows the number of unique and common InDels. The dots represent the presence of the InDels associated with the trait that is listed on the left side. The vertical column with only one dot represents unique InDels in one trait. The vertical column with more than one dot represents the common InDels for multiple traits.

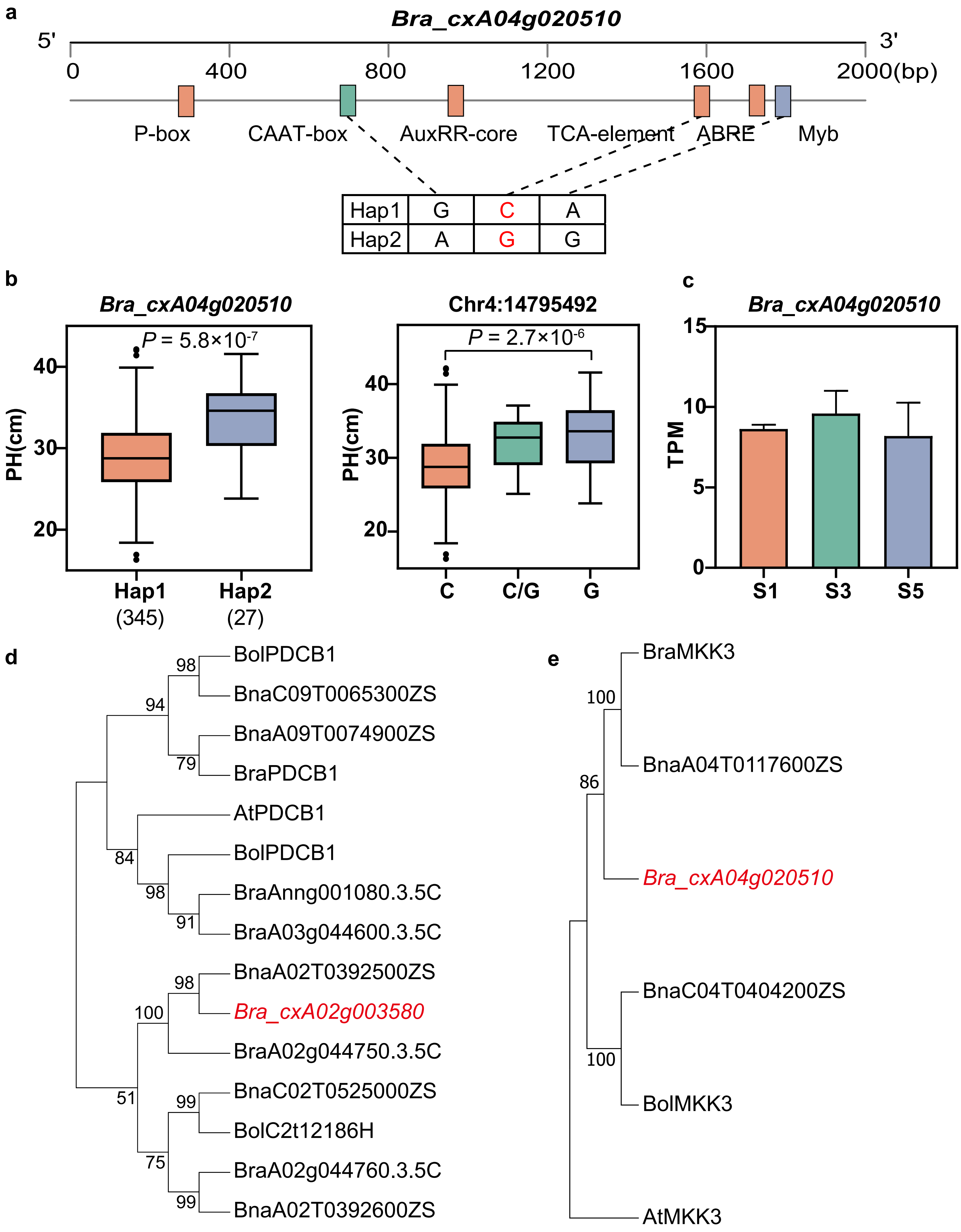

Supplementary Figure 11. Identification of the candidate gene *Bra_cxA04g020510* for Plant height. a, Promoter element and DNA polymorphism of *Bra_cxA04g020510*. b, Boxplot of PH for the haplotypes (Hap) of *Bra_cxA04g020510*. Center line, median, box limits, upper and lower quartiles; whiskers, 1.5× the interquartile range; and dots represent outliers. Significant differences between the haplotypes were evaluated by a two-tailed t-test and shown by *P* value or different letters ($P < 0.05$). c, Expression levels of *Bra_cxA04g020510* in different stages of stalks based on TPM from RNA-seq results. S1, S3, and S5 represent the seedling stage, the bolting stage, and the flowering or harvesting stage, respectively. d, e, Phylogenetic trees constructed with neighbor joining analysis to compare with Other *Brassica* plants and *Arabidopsis*.

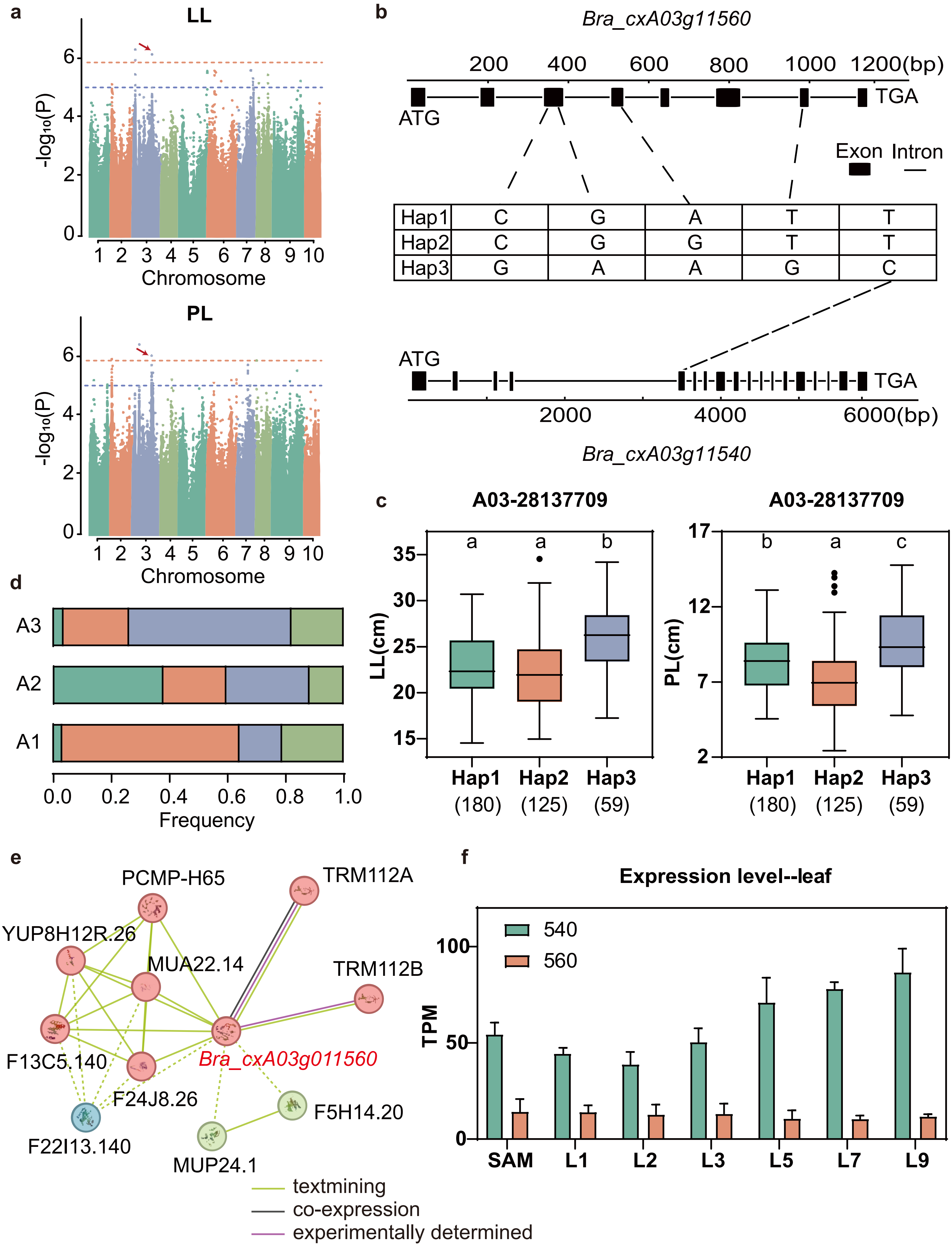

Supplementary Figure 12. GWAS for Leaf length, Petiole length, and identification of the candidate gene *Bra_cxA03g011560* and *Bra_cxA03g011540*. a, Manhattan plots showed the locus (A03:28137709) for LL, PL based on SNP-GWAS. b, Exon-intron structure and DNA polymorphism of *Bra_cxA03g011560* and *Bra_cxA03g011540*. c, Boxplot of LL, PL for the haplotypes (Hap) of *Bra_cxA03g011560* and *Bra_cxA03g011540*. Center line, median, box limits, upper and lower quartiles; whiskers, 1.5× the interquartile range; and dots represent outliers. Significant differences between the haplotypes were evaluated by a two-tailed t-test and shown by *P* value or different letters ($P < 0.05$). d, Frequency changes of *Bra_cxA03g011560* and *Bra_cxA03g011540* haplotypes in different breeding eras. e, Protein-protein interaction network analysis of gene *Bra_cxA03g011560*. f, Expression levels of *Bra_cxA03g011560* and *Bra_cxA03g011540* in different stages of leaf based on TPM from RNA-seq results.

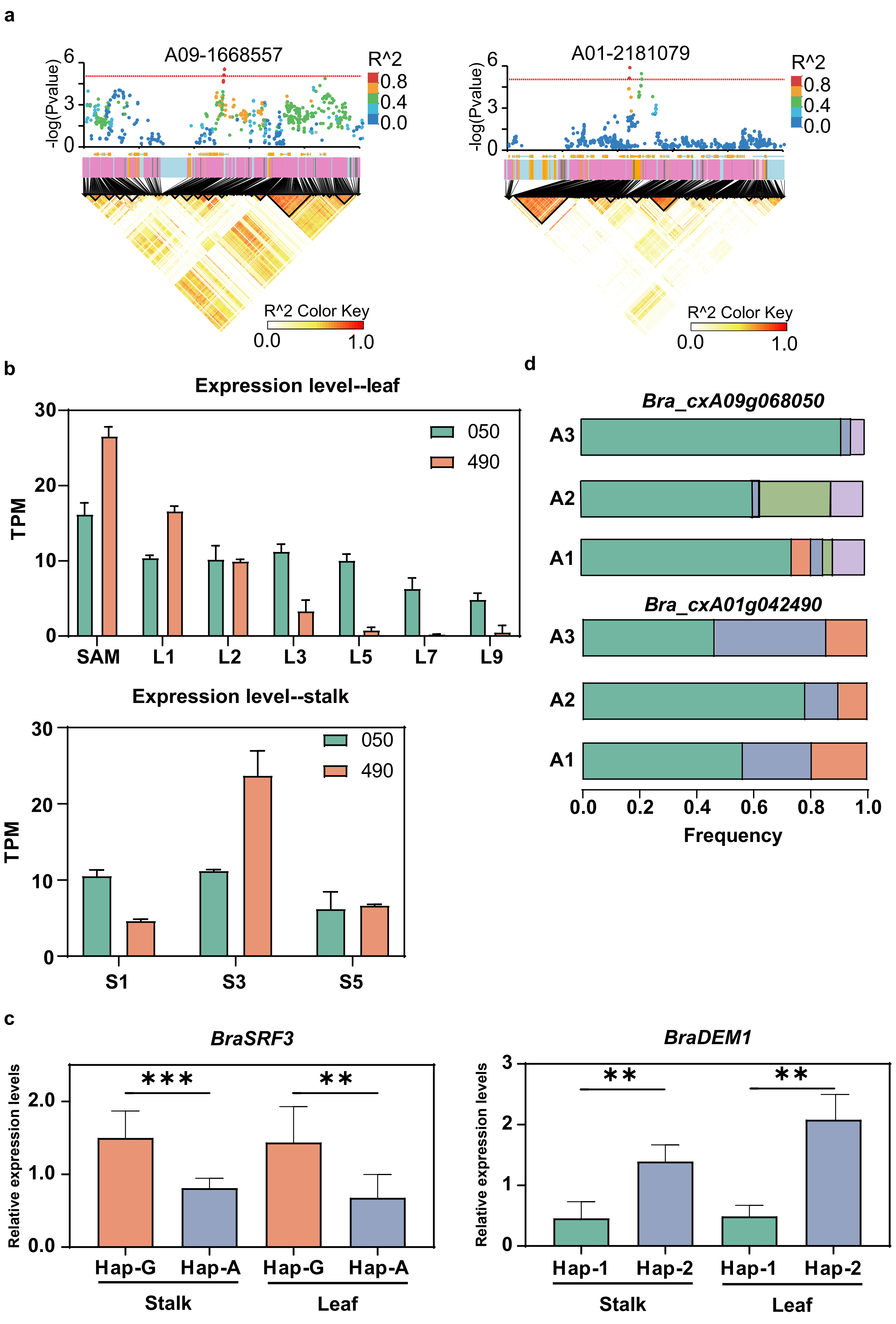

Supplementary Figure 13. Identification of the candidate genes *Bra_cxA09g068050* and *Bra_cxA01g042490*. a, Local Manhattan plot (top), gene models (middle), and LD heatmap (bottom) surrounding the A09-1668557 and A01-2181079. The horizontal dashed line represents the significance threshold ($P < 1 \times 10^{-5}$). b, Expression levels of *Bra_cxA09g068050* and *Bra_cxA01g042490* in different stages of leaf and stalk based on TPM from RNA-seq results. S1, S3, and S5 represent the seedling stage, the bolting stage, and the flowering or harvesting stage, respectively. c, Genes correspond to the relative expression levels of different haplotypes at the bolting stage. Significant differences were evaluated by two-tailed Student's t-test (** P < 0.01, *** P < 0.001). d, Frequency changes of *Bra_cxA09g068050* and *Bra_cxA01g042490* haplotypes in different breeding periods.

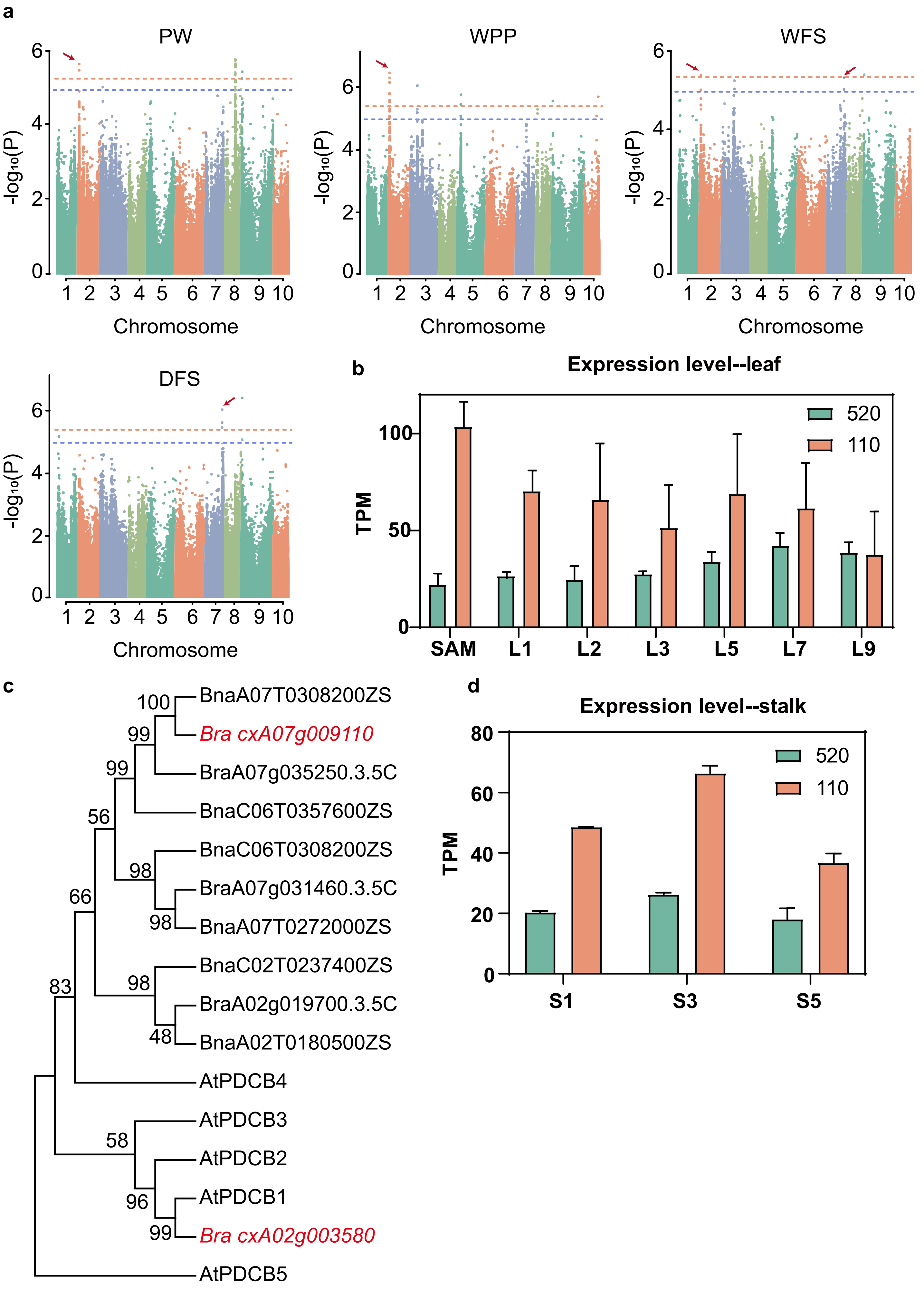

Supplementary Figure 14. GWAS for PW, WPP, WFS, DFS, and identification of the candidate gene *Bra_cxA02g046520* and *Bra_cxA07g009110.* a, Manhattan plots showed the locus (A02-1569970, A07-24297027) for PW, WPP, WFS, DFS based on SNP-GWAS. b, d, Expression levels of *Bra_cxA02g046520* and *Bra_cxA07g009110* in different stages of leaf and stalk based on TPM from RNA-seq results. S1, S3, and S5 represent the seedling stage, the bolting stage, and the flowering or harvesting stage, respectively. c, Phylogenetic trees constructed with neighbor joining analysis to compare with Other *Brassica* plants and *Arabidopsis*.

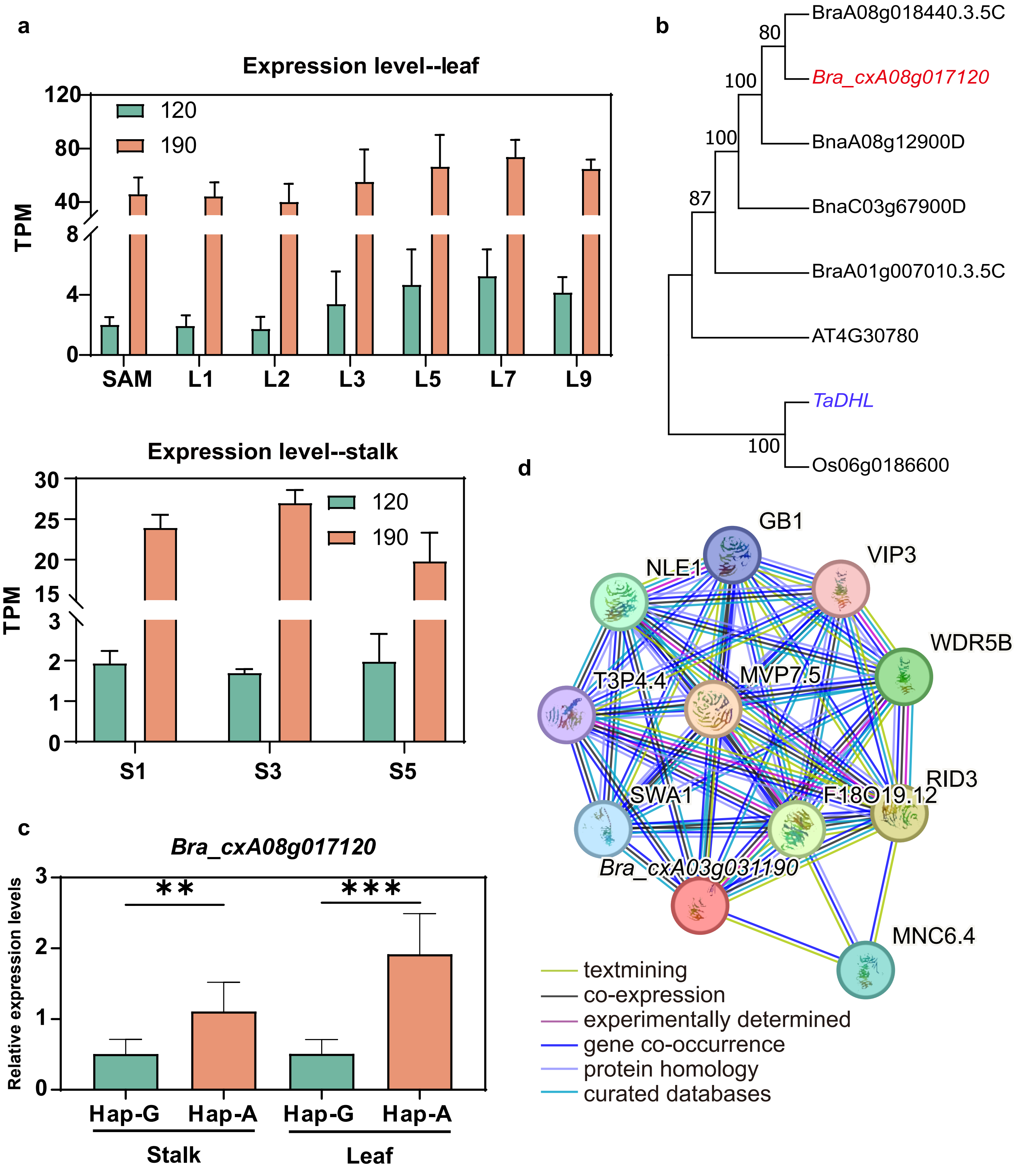

Supplementary Figure 15. Expression profiling, protein-protein interactions, and evolutionary analysis of the candidate gene *Bra_cxA08g017120* and *Bra_cxA03g031190*. a, Expression levels of *Bra_cxA08g017120* and *Bra_cxA03g031190* in different stages of leaf and stalk based on TPM from RNA-seq results. S1, S3, and S5 represent the seedling stage, the bolting stage, and the flowering or harvesting stage, respectively. b, Phylogenetic trees constructed with neighbor joining analysis to compare with Other *Brassica* plants, *Arabidopsis*, and wheat. c, Genes correspond to the relative expression levels of different haplotypes at the bolting stage. Significant differences were evaluated by two-tailed Student's t-test (** $P < 0.01$, *** $P < 0.001$). d, Protein-protein interaction network analysis of gene *Bra_cxA03g031190*.

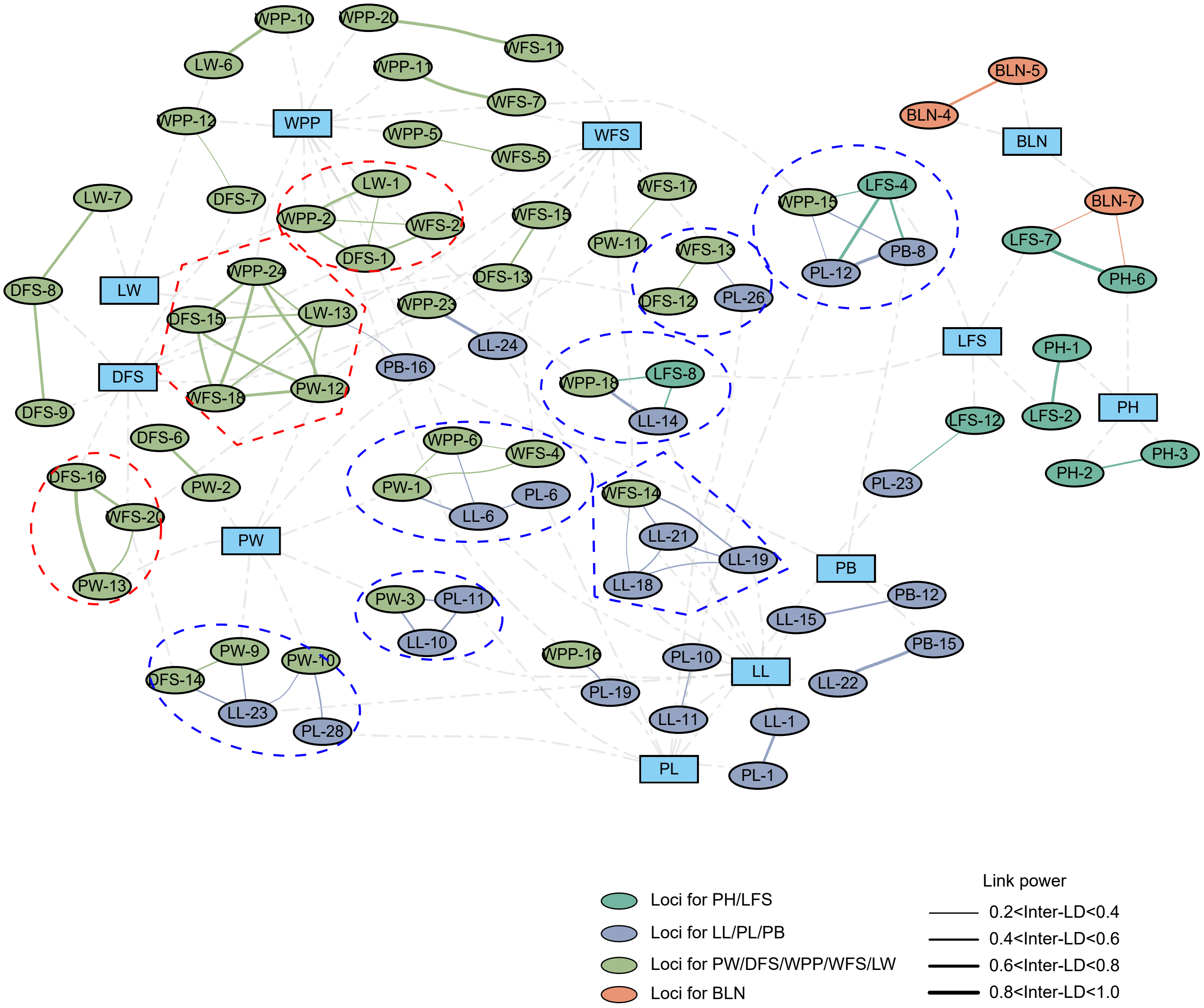

Supplementary Figure 16. Association networks across different traits in flowering Chinese cabbage. Network for 11 traits based on the link powers between loci. The nodes represent traits and their corresponding loci (see Supplementary Table 13). The edges between the loci from different traits are linked by LD. Only the edges with an average LD ≥ 0.2 are shown.The red dotted circle emphasizes the association between traits in the same group, and the blue dotted circle emphasizes the association between traits in different groups.
